# Tianqi Jiangtang Capsule in the treatment of patients with diabetes: a systematic review and meta-analysis

**DOI:** 10.3389/fphar.2025.1719112

**Published:** 2026-01-21

**Authors:** Yanjiao Liu, Yifan Chen, Liuding Wang, Zhonghui Jiang, Zhuye Gao

**Affiliations:** 1 Department of Cardiology, Xiyuan Hospital, China Academy of Chinese Medical Sciences, Beijing, China; 2 Department of Neurology, Xiyuan Hospital, China Academy of Chinese Medical Sciences, Beijing, China; 3 Department of Geriatrics, Eye Hospital, China Academy of Chinese Medical Sciences, Beijing, China

**Keywords:** traditional Chinese medicine, diabetes mellitus, hemoglobin, fasting plasmaglucose, 2 h postprandial glucose

## Abstract

**Background:**

Tianqi Jiangtang Capsule (TJC) is a commercial Chinese polyherbal preparation (CCPP) commonly used as adjunctive therapy for glucose management in diabetes. While its potential multi-system effects have been observed, a systematic evaluation focusing on glycemic control remains limited. This study aims to primarily assess the glucose-lowering efficacy of TJC in diabetic patients.

**Materials and Methods:**

We conducted a comprehensive search for relevant randomized controlled trials (RCTs) across nine electronic databases from their inception to 1 September 2025. Two independent reviewers performed trial selection, data extraction, and risk-of-bias assessment. Meta-analyses of efficacy and safety outcomes were performed using RevMan 5.3 and Stata 17. Evidence quality was evaluated using GRADE methodology.

**Results:**

13 RCTs involving 1,298 diabetic patients were included. Compared with conventional treatment (CT) alone or combined with placebo, TJC plus CT significantly improved primary glycemic parameters: glycated hemoglobin (mean difference [*MD*] = −1.22, 95% confidence interval [*CI*] −1.70 to −0.74, *P* < 0.01), fasting blood glucose (*MD* = −1.37, 95% *CI* -1.74 to −0.99, *P* < 0.01), and 2 h postprandial blood glucose (*MD* = −2.07, 95% *CI* -2.56 to −1.58, *P* < 0.01). As exploratory findings, TJC also demonstrated beneficial effects on lipid profiles, inflammatory markers, and renal function. No significant difference was observed in the incidence of adverse events between groups.

**Conclusion:**

TJC significantly improves glycemic control in patients with diabetes and shows potential multi-system benefits. However, its efficacy and safety profile require further validation in large-scale, high-quality trials, particularly regarding the influence of genetic factors on treatment response.

## 1 Introduction

Diabetes is a chronic disease mainly characterized by elevated blood sugar levels. This condition results from an absolute or relative insufficiency of insulin secretion, or an impaired utilization of insulin (Singh et al., 2025). It is estimated that by 2050, more than 1.31 billion people will have diabetes (GBD, 2023). The long-term management of diabetes continues to pose challenges due to limitations in current drug therapies. Particularly in type 2 diabetes, oral hypoglycemic agents often fail to achieve adequate glycemic control (Best et al., 2012). Moreover, with the exception of SGLT2 inhibitors, most oral hypoglycemic agents demonstrate no definitive benefits for patients’ cardio-renal outcomes. Some may even exacerbate conditions such as heart failure (Endocrinology, 2019). Exogenous insulin therapy remains essential in diabetes management. However, its use is associated with hypoglycemia risks and potential cardiovascular concerns (Peng et al., 2018; Kenny and Abel, 2019). Therefore, there is an urgent need for multifaceted glucose-lowering strategies that extend beyond glycemic control alone.

Traditional Chinese medicine therapy has unique therapeutic effects in preventing and treating diabetes and its complications (Li and Zhao, 2024). Tianqi Jiangtang Capsule (TJC) is a commercial Chinese polyherbal preparation (CCPP) widely used for patients with type 2 diabetes. It is the main intervention drug for the “11 th Five-Year Plan” science and technology project “Research on Traditional Chinese Medicine Treatment of Prediabetes Type 2”(He et al., 2018). Numerous studies have shown that TJC not only delays the progression of prediabetes, but also exerts a significant therapeutic effect in patients with diabetes, diabetes-related cerebrovascular disease and diabetic nephropathy (Cao et al., 2015; Chai et al., 2017; Hou G., 2017; Sun et al., 2019). However, the current evidence regarding the efficacy of TJC in diabetic patients predominantly relies on earlier clinical studies. They are considerably outdated compared to contemporary therapeutic standards. Our study therefore conducts an updated systematic review of TJC, incorporating recent clinical findings to comprehensively reassess its efficacy and safety profile.

## 2 Materials and methods

The protocol was registered in PROSPERO (CRD420251137368). Our study adhered to the Preferred Reporting Items for Systematic Reviews and Meta-Analyses 2020 statement (Page et al., 2021).

Tianqi Jiangtang Capsule (TJC) is a Chinese patent medicine approved by the China National Medical Products Administration (NMPA, 2009). It is composed of ten botanical drugs: *Astragalus membranaceus* Fisch. ex Bunge [Fabaceae; Astragali Radix], *Trichosanthes kirilowii* Maxim. [Cucurbitaceae; Trichosanthis Radix], *Ligustrum lucidum* W.T.Aiton [Oleaceae; Ligustri Lucidi Fructus], *Dendrobium nobile* Lindl. [Orchidaceae; Dendrobii Caulis], *Panax ginseng* C.A.Mey. [Araliaceae; Ginseng Radix et Rhizoma], *Lycium chinense* Mill. [Solanaceae; Lycii Cortex], *Coptis chinensis* Franch. [Ranunculaceae; Coptidis Rhizoma], *Cornus officinalis* Siebold & Zucc. [Cornaceae; Corni Fructus], *Eclipta prostrata* (L.) L. [Asteraceae; Ecliptae Herba], *Rhus chinensis* Mill. [Anacardiaceae; Galla Chinensis]. All botanical drugs were verified using Plants of the World Online (POWO). We followed the ConPhyMP consensus reporting guidelines (Heinrich et al., 2022) and completed the ConPhyMP preparation as detailed in Supplementary null Appendix 1. A comprehensive summary of the botanical drugs compositions reported in the studies included in our meta-analysis is provided in Supplementary Table S3.

### 2.1 Inclusion and exclusion criteria

#### 2.1.1 Inclusion and exclusion criteria based on the PICOS framework

##### 2.1.1.1 Inclusion criteria

Population: Patients with a confirmed diagnosis of diabetes mellitus or those meeting the diagnostic criteria outlined in the 2024 American Diabetes Association’s Standards of Care in Diabetes (2024). Intervention: The control group received conventional therapy alone or in combination with a placebo. Conventional therapy may include treatments tailored to the patient’s underlying conditions, such as glucose-lowering or lipid-lowering agents. The observation group received TJCs in addition to the control regimen. Primary outcomes: Glycated hemoglobin (HbA1c), fasting plasma glucose (FPG), and 2 h postprandial glucose (2hPG); Secondary outcomes: High-sensitivity C-reactive protein (hs-CRP), tumor necrosis factor-α (TNF-α), interleukin-6 (IL-6), total cholesterol (TC), triglycerides (TG), low-density lipoprotein cholesterol (LDL-C), blood urea nitrogen (BUN), and serum creatinine (Scr); Safety outcomes: Adverse reactions (AR). Study Design: Randomized controlled trials (RCTs).

##### 2.1.1.2 Exclusion criteria

Non-randomized controlled trials; Studies for which the full text could not be retrieved; Studies with incomplete, seriously erroneous, or biased data; Duplicated publications.

### 2.2 Search strategy

We conducted a computerized search of the following databases: China National Knowledge Infrastructure, WanFang, Chinese BioMedical Literature Database, China Science and Technology Journal Database, PubMed, Cochrane Library, ClinicalTrials.gov, Web of Science, and EMBASE. Studies on TJC for the treatment of patients with diabetes were collected. The search period spanned from the establishment of each database to 1 September 2025. Search terms included: “tianqijiangtang capsule”, “tianqijiangtang”, “tianqi jiangtang”, “tianqi”, “tianqi hypoglycemic capsule”, “Diabetes Mellitus”, “Diabetes Insipidus”, “Diabetic Diet”, “Gastroparesis”, “Glucose Intolerance”, “Advanced Glycation End Products”, “Prediabetic State”, and “Scleredema Adultorum” (Supplementary Table S1).

### 2.3 Study selection and data extraction

Two researchers (Liu Yanjiao and Chen Yifan) independently reviewed the titles and abstracts of each study. They then performed a full-text review of articles that were potentially eligible for inclusion in the meta-analysis. Literature screening and data extraction were conducted according to predefined eligibility criteria, after which the results were cross-verified. Any discrepancies were resolved through discussion or by consultation with a third researcher. Data were extracted using a standardized Excel form (Table 1), including, but not limited to: author(s), publication year, sample size, interventions, outcome measures, and participants’ baseline characteristics.

**TABLE 1:** Basic characteristics of included trials.

Study	Publication year	Sample size	Male/Female		Age (year)		Diabetes duration		BMI(kg/m^2^)		Intervention		Duration	Outcomes
		E/C	E	C	E	C	E	C	E	C	E	C		
Ma et al. (2021)	2021	67/67	35/32	37/30	31–67 (54.89 ± 1.76)	31–66 (54.57 ± 1.49)	1–11 (7.457 ± 1.36)	1–11 (7.29 ± 1.18)	−/−	−/−	(1)Tianqi Jiangtang capsules 1.6 g three times daily. (2)CTs including Dapagliflozin 10 mg once daily	(1)CTs	8 weeks	①②③⑫
Shen (2019)	2019	36/36	20/16	21/15	43–80 (65.11 ± 6.85)	44–79 (63.45 ± 6.81)	3–13 (6.76 ± 0.71)	3–14 (6.73 ± 0.69)	21–27 (24.55 ± 2.19)	20–28 (24.34 ± 2.13)	(1)Tianqi Jiangtang capsules 1.6 g three times daily. (2)CTs including calcium dobesilate 0.5 g three times daily, blood glucose control, lipid regulation and blood pressure reduction	(1)CTs	12 weeks	①②③⑩⑪
Yang et al. (2019)	2019	42/42	25/17	22/20	39–70 (53.1 ± 7.0)	37–69 (52.3 ± 7.5)	0.75–5 (3.5 ± 1.1)	0.5–5 (3.2 ± 1.4)	20.5–30.9 (25.3 ± 1.8)	20.9–31.6 (25.9 ± 2.3)	(1)Tianqi Jiangtang capsules 1.6 g three times daily. (2)CTs including saxagliptin 5 mg once daily, medical nutrition therapy, body weight control, monitoring blood glucose, health education and exercise guidanceetc.	(1)CTs	8 weeks	①②③⑤⑥⑫
Qiao (2019)	2019	51/51	27/24	28/23	62.64 ± 5.82	62.98 ± 6.02	7.28 ± 2.14	7.19 ± 2.24	−/−	−/−	(1)Tianqi Jiangtang capsules 1.6 g three times daily. (2)CTs including calcium dobesilate 0.5 g three times daily and other basic treatment for diabetic nephropathy	(1)CTs	8 weeks	⑦⑧⑩⑪⑫
Xu et al. (2018)	2018	36/36	21/15	17/19	49–70 (59.1 ± 5.4)	47–69 (60.2 ± 5.1)	6–14 (9.2 ± 2.6)	5–15 (9.5 ± 2.3)	−/−	−/−	(1)Tianqi Jiangtang capsules 1.6 g three times daily. (2)CTs including losartan potassium 100 mg once daily and other basic treatment	(1)CTs	12 weeks	①④⑤⑥⑩⑪⑫
Chai et al. (2017)	2017	48/48	30/18	28/20	65.47 ± 4.13	66.00 ± 4.25	6.59 ± 2.41	6.60 ± 2.36	24.57 ± 2.64	24.82 ± 1.95	(1)Tianqi Jiangtang capsules 1.6 g three times daily. (2)CTs including metformin tables 500 mg three times daily, psychologcal counseling, health education and exercise guidance	(1)CTs	4 weeks	①②③④⑦⑧⑨⑫
Hou G.((2017)	2017	48/47	26/22	27/20	47–78 (63.12 ± 4.42)	48–79 (63.45 ± 4.53)	5–9 (7.11 ± 2.38)	5–10 (7.26 ± 2.41)	−/−	−/−	(1)Tianqi Jiangtang capsules 1.6 g three times daily. (2)CTs including metformin tables 500 mg twice daily	(1)CTs	8 weeks	⑩⑪
Hou C. (2017)	2017	49/49	25/24	26/23	49.8 ± 6.9	50.2 ± 7.3	−/−	−/−	−/−	−/−	(1)Tianqi Jiangtang capsules 1.6 g three times daily. (2)CTs including administering subcutaneous insulin injections or oral reglazone tablets to keep blood glucose within the target range, oral ACEIs or ARBs tablets to control blood pressure within 130/80 mmHg for patients with hypertension, adopting a low-fat deit and administering statins for patients with hyperlipidemia, diet control, health education and exercise guidance	(1)CTs	8 weeks	①②③⑦⑧⑨⑩⑪⑫
Wu et al. (2017)	2017	72/72	40/32	43/29	45–74 (58.4 ± 6.8)	46–75 (57.7 ± 6.3)	3–15 (6.8 ± 1.5)	3–14 (6.2 ± 1.3)	20–28 (23.9 ± 3.6)	20–27 (23.6 ± 3.1)	(1)Tianqi Jiangtang capsules 1.6 g three times daily. (2)CTs including calcium dobesilate 0.5 g three times daily, blood glucose control, lipid regulation, blood pressure reduction and improving microcirculation	(1)CTs	8 weeks	④⑥⑩⑪⑫
Wu and Gao, (2017)	2017	30/30	16/14	18/12	58.94 ± 5.36	58.17 ± 4.89	8.44 ± 2.25	8.69 ± 2.48	22.36 ± 4.65	22.36 ± 4.65	(1)Tianqi Jiangtang capsules 1.6 g three times daily	(1)Placebo	16 weeks	①⑦⑧⑨⑩⑪
Tang et al. (2016)	2016	54/54	34/20	31/23	58.34 ± 8.03	59.47 ± 7.45	11.97 ± 2.60	12.58 ± 2.49	−/−	−/−	(1)Tianqi Jiangtang capsules 1.6 g three times daily. (2)CTs including administering subcutaneous insulin injections or oral reglazone tablets to keep blood glucose within the target range (FPG<6.1 mmol/L, 2hPG<8.3 mmol/L), oral fosinopril sodium tablets or irbesartan tablets to control blood pressure within 130/85 mmHg for patients with hypertension, diet control and exercise therapy	(1)CTs	8 weeks	①②③⑦⑧⑨⑩⑪⑫
Cao et al. (2015)	2015	40/40	19/19	19/17	33–63 (50.08 ± 7.21)	34–62 (49.69 ± 7.12)	1–7 (4.08 ± 1.58)	1–7 (4.14 ± 1.55)	24.08 ± 2.87	23.83 ± 2.87	(1)Tianqi Jiangtang capsules 1.6 g three times daily. (2)CTs including metformin tables 500 mg three times daily , diet control and exercise therapy	(1)CTs	8 weeks	①③④⑤⑥
Lian et al. (2011)	2011	40/39	−/−	−/−	−/−	−/−	−/−	−/−	−/−	−/−	(1)Tianqi Jiangtang capsules 1.6 g three times daily. (2)CTs including metformin tables 500 mg three times daily	(1)Placebo (2)CTs	12 weeks	①②③⑦⑧⑨

Abbreviation: CTs, conventional treatments; E, experiment group; C, control group; ACEIs, angiotensin-converting enzyme inhibitors; ARBs, angiotensin receptor blockers; ① HbA1c; ②FPG; ③ 2hPG; ④ hs-CRP; ⑤ IL-6; ⑥ TNF-α; ⑦ TC; ⑧ TG; ⑨ LDL-C; ⑩ BUN; ⑪ SCr; ⑫ adverse reactions.

### 2.4 Risk of bias assessment

The risk of bias for each outcome was assessed using the Cochrane Risk of Bias tool (ROB2). Based on the descriptions provided in the articles regarding random sequence generation, allocation concealment, blinding, and other criteria, each included study was judged as having either a low, high, or unclear risk of bias.

### 2.5 Data analysis

Meta-analysis was performed using RevMan software (version 5.3) and Stata 17. For continuous variables measured with the same unit, the mean difference (*MD*) was used as the effect measure; for those with different units, the standardized mean difference (*SMD*) was applied. For dichotomous variables, the risk ratio (RR) was selected to summarize the results. All outcomes were expressed as effect estimates with their corresponding 95% confidence intervals (*CI*).

Heterogeneity among the included studies was assessed using the *I* ^*2*^ statistic. A fixed-effect model was applied when heterogeneity was not significant (*I* ^*2*^ < 50%), whereas a random-effect model was used when substantial heterogeneity was present (*I* ^*2*^ ≥ 50%), and the sources of heterogeneity were further explored. A significance level of α = 0.05 was set for the meta-analysis. We performed subgroup analyses to evaluate the impact of between-study heterogeneity, such as differences in follow-up duration, on the overall results. Additionally, sensitivity analysis was conducted to explore potential sources of statistical heterogeneity and to examine the robustness of the findings. If more than ten studies were included for a given outcome, potential publication bias was assessed by visually inspecting funnel plots and statistically using Begg’s and Egger’s tests.

### 2.6 Certainty assessment

Two independent reviewers (Liu Yanjiao and Jiang Zhonghui) rated the certainty of the evidence. The assessment used the GRADE framework. It covered risk of bias, imprecision, inconsistency, indirectness, and publication bias.

## 3 Results

### 3.1 Study selection

**FIGURE 1:**
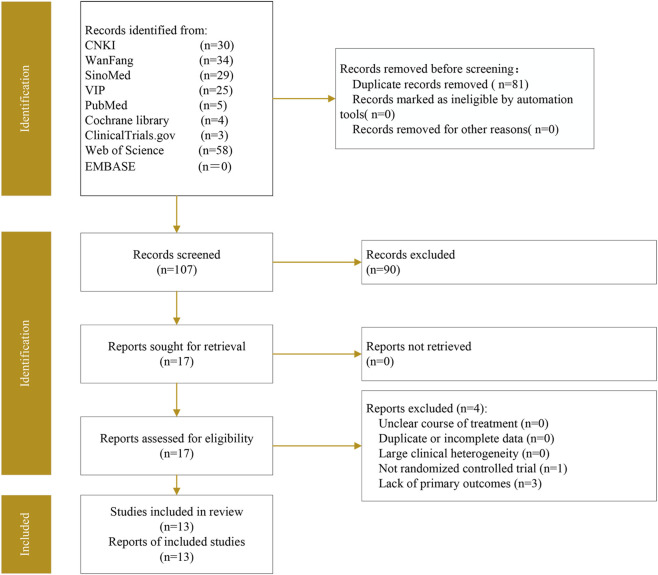
The preferred reporting items for systematic reviews and meta-analyses flow diagram for study selection.

A total of 188 records were identified through database searches (CNKI n = 30, WanFang n = 34, SinoMed n = 29, VIP n = 25, PubMed n = 5, Cochrane Library n = 4, ClinicalTrials.gov n = 3, Web of Science n = 58, EMBASE n = 0) (Figure 1). After 81 duplicates were removed by using EndNote 20, we screened the titles and abstracts, and excluded an additional 90 records that did not meet the eligibility criteria. The remaining 17 records underwent full-text review. Among these, four studies were excluded due to reasons such as non-randomized controlled trials or lack of relevant outcome measures (Supplementary Table S2). Ultimately, 13 articles were included in the quantitative synthesis (Figure 1).

### 3.2 Study characteristics

A total of 13 randomized controlled trials (Lian et al., 2011; Cao et al., 2015; Tang et al., 2016; Chai et al., 2017; Hou G., 2017; Hou C., 2017; Wu et al., 2017; Wu and Gao, 2017; Xu et al., 2018; Qiao, 2019; Shen, 2019; Yang et al., 2019; Ma et al., 2021) (RCTs) investigating TJC in the treatment of patients with diabetes were included. All studies were published in Chinese between 2011 and 2021, with a total sample size of 1,298 participants—650 in the intervention group and 648 in the control group. The smallest sample size was 60, and the largest was 144 (Table 1).

### 3.3 Methodological quality

**FIGURE 2:**
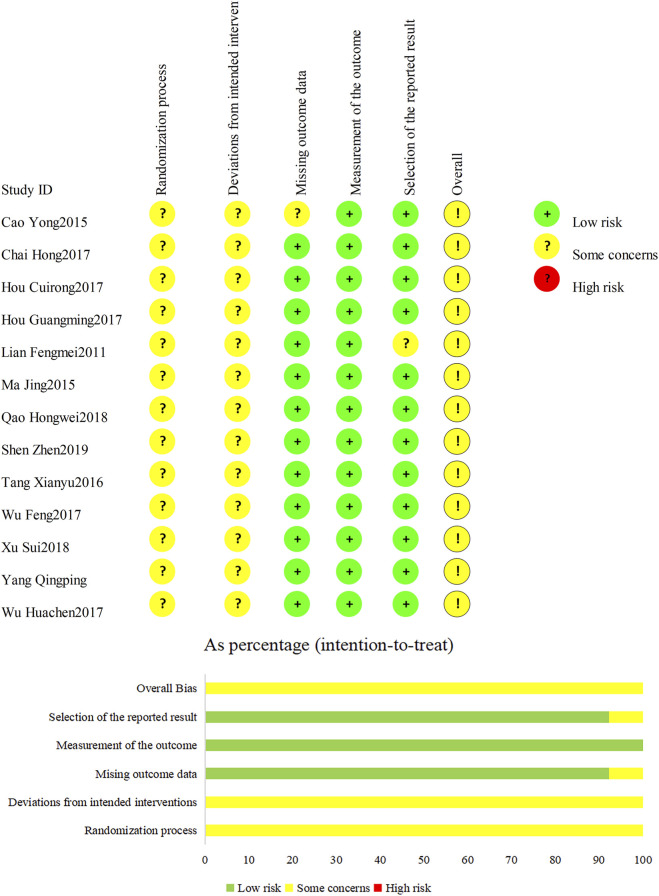
Risk of bias graph: judgments about each risk of bias item presented across all included trials.

The risk of bias in the included studies was assessed using the Cochrane ROB2 tool (Figure 2). Among the included studies, eight studies described the use of a random number table for allocation, while six only mentioned the term “randomized” without specifying the method. None of the included studies reported blinding or allocation concealment procedures. Lian et al. (2011) reported no pre-specified adverse reactions or adverse events, though all other trials included these outcomes. Two studies documented loss to follow-up and withdrawals. Cao et al. (2015) reported “4 cases lost to follow-up in the control group and 2 cases lost to follow-up in the treatment group”. Lian et al. (2011) noted that “2 participants dropped out, and 77 completed the study” (Figure 2).

### 3.4 Primary outcomes

#### 3.4.1 HbA1c

**FIGURE 3:**
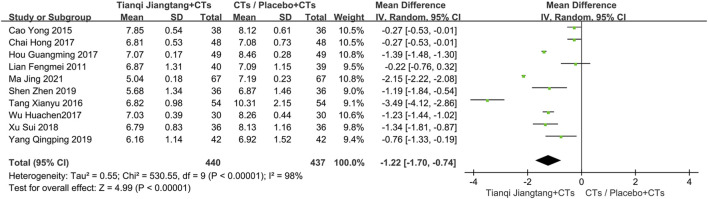
Overall pooled results forest plot comparing Tianqi Jiangtang Capsule plus conventional treatments (CTs) to placebo plus CTs or CTs alone on HbA1c.

A total of 10 studies (Lian et al., 2011; Cao et al., 2015; Tang et al., 2016; Chai et al., 2017; Hou C., 2017; Wu and Gao, 2017; Xu et al., 2018; Shen, 2019; Yang et al., 2019; Ma et al., 2021) reported HbA1c levels (Figure 3), with significant heterogeneity observed among them (*P* < 0.00001, *I* ^*2*^ = 98%). Sensitivity analysis indicated that the results were robust. A random-effects model was therefore applied for meta-analysis, demonstrating significantly lower HbA1c levels in the observation group compared to the control group (*MD* = −1.22, 95% *CI* = −1.70 to −0.74, *P* < 0.00001) (Table 2).

**TABLE 2:** Overall pooled results of the meta-analysis.

Outcomes	Number of included studies	Heterogeneity test		Effect model	Results		
		*I* ^*2*^ (%)	*P*		*MD/RR*	(95%*CI*)	*P*
Primary outcomes							
HbA1c	10	98	P < 0.00001	Random-effect model	−1.22	−1.70–0.74	P < 0.00001
FPG	8	94	P < 0.00001	Random-effect model	−1.37	−1.74–0.99	P < 0.00001
2hPG	8	94	P < 0.00001	Random-effect model	−2.07	−2.56–1.58	P < 0.00001
Secondary outcomes							
hs-CRP	3	96	P < 0.00001	Random-effect model	−2.51	−3.71–1.30	P < 0.0001
IL-6	3	0	P = 0.93	Fixed-effect model	−3.43	−3.87–2.98	P < 0.00001
TNF-α	4	97	P < 0.00001	Random-effect model	−7.66	−11.26–4.06	P < 0.0001
TC	6	87	P < 0.00001	Random-effect model	−0.52	−0.70–0.35	P < 0.00001
TG	6	76	P = 0.001	Random-effect model	−0.24	−0.34–0.15	P < 0.00001
LDL-C	5	94	P < 0.00001	Random-effect model	−0.94	−1.18–0.71	P < 0.00001
BUN	7	89	P < 0.00001	Random-effect model	−0.96	−1.17–0.76	P < 0.00001
Scr	7	97	P < 0.00001	Random-effect model	−18.53	−23.78–13.28	P < 0.00001

**FIGURE 4:**
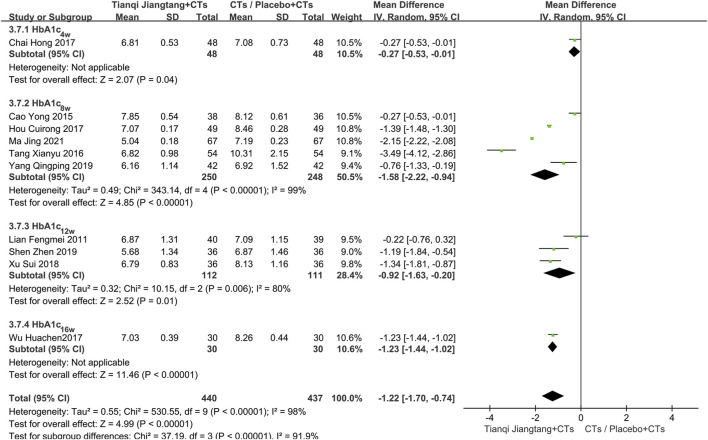
Subgroup analysis forest plot comparing Tianqi Jiangtang Capsule plus conventional treatments (CTs) to placebo plus CTs or CTs alone on HbA1c.

Subgroup analysis based on intervention duration was performed. One study (Chai et al., 2017) with a 4 week intervention showed a significant difference in efficacy between the two groups (*MD* = −0.27, 95% *CI* = −0.53 to −0.01, *P* = 0.04). Five studies (Cao et al., 2015; Tang et al., 2016; Hou C., 2017; Yang et al., 2019; Ma et al., 2021) with an 8 week intervention exhibited significant heterogeneity (*P* < 0.01, *I* ^*2*^ = 99%). Differences in study populations and intervention methods across these trials were identified as potential sources of heterogeneity. Using a random-effects model, the pooled effect size indicated significantly lower HbA1c levels in the observation group (*MD* = −1.58, 95% *CI* = −2.22 to −0.94, *P* < 0.00001). Three studies (Lian et al., 2011; Xu et al., 2018; Shen, 2019) with a 12 week intervention showed substantial heterogeneity (*P* < 0.01, *I* ^*2*^ = 80%), and thus a random-effects model was used. The combined results demonstrated a significant difference between the groups (*MD* = −0.92, 95% *CI* = −1.63 to −0.20, *P* = 0.01). Excluding the study by Lian et al. (2011), between-study heterogeneity decreased significantly (*P* = 0.71, *I* ^*2*^ = 0%). A full-text review revealed that the study by Lian et al. (2011) involved patients with diabetes, while the other two studies focused on patients with diabetic kidney disease, suggesting that differences in study population may account for the heterogeneity. One study (Wu and Gao, 2017) with a 16 week intervention demonstrated a more pronounced reduction in HbA1c levels in the observation group (*MD* = −1.23, 95% *CI* = −1.44 to −1.02, *P* < 0.00001) (Figure 4).

#### 3.4.2 FPG

**FIGURE 5:**
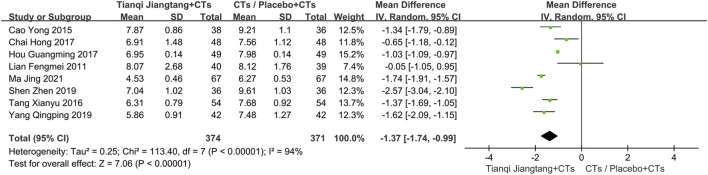
Overall pooled results forest plot comparing Tianqi Jiangtang Capsule plus conventional treatments (CTs) to placebo plus CTs or CTs alone on FPG.

A total of eight studies (Lian et al., 2011; Cao et al., 2015; Tang et al., 2016; Chai et al., 2017; Hou C., 2017; Shen, 2019; Yang et al., 2019; Ma et al., 2021) reported FPG levels (Figure 5), with significant heterogeneity among them (*P* < 0.00001, *I* ^*2*^ = 94%). Sensitivity analysis suggested that the results were robust. A random-effects model was therefore employed for meta-analysis, which indicated that the observation group had significantly lower FPG levels than the control group after treatment (*MD* = −1.37, 95% *CI* = −1.74 to −0.99, *P* < 0.00001) (Table 2).

**FIGURE 6:**
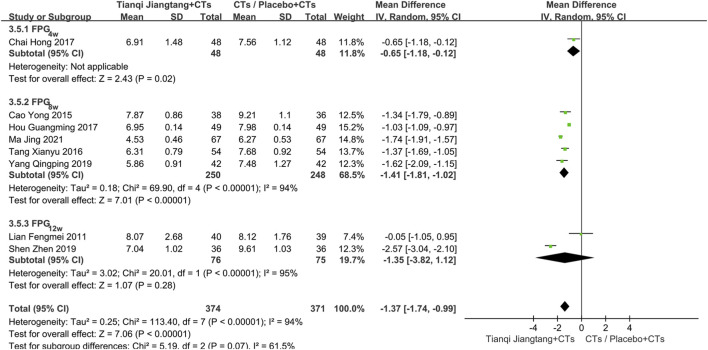
Subgroup analysis forest plot comparing Tianqi Jiangtang Capsule plus conventional treatments (CTs) to placebo plus CTs or CTs alone on FPG.

Subgroup analysis based on intervention duration was conducted. One study (Chai et al., 2017) with a 4 week intervention period showed a statistically significant difference between the two groups (*MD* = −0.65, 95% *CI* = −1.18 to −0.12, *P* = 0.02). In the five studies (Cao et al., 2015; Tang et al., 2016; Hou C., 2017; Yang et al., 2019; Ma et al., 2021) with an 8 week intervention, significant heterogeneity was observed (*P* < 0.01, *I* ^*2*^ = 94%). After excluding the study by Hou G. (2017) (Hou C., 2017), the heterogeneity decreased substantially (*P* = 0.13, *I* ^*2*^ = 48%). A full-text review indicated that the study by Hou G. (2017) involved patients with early-stage diabetes, while the other four studies included patients who met diagnostic criteria for diabetes (with one study specifically focusing on diabetic kidney disease). Differences in study populations were identified as a potential source of heterogeneity. Using a random-effects model, the pooled effect size showed significantly lower FPG levels in the observation group (*MD* = −1.41, 95% *CI* = −1.81 to −1.02, *P* < 0.00001). Two studies (Lian et al., 2011; Shen, 2019) with a 12 week intervention exhibited significant heterogeneity (*P* < 0.01, *I* ^*2*^ = 95%). A random-effects model was applied, and the meta-analysis results showed no significant difference in efficacy between the two groups (*MD* = −1.35, 95% *CI* = −3.82 to 1.12, *P* = 0.28). After careful review of the full texts, it was found that the included populations differed between the two studies, which may explain the observed heterogeneity (Figure 6).

#### 3.4.3 2hPG

**FIGURE 7:**
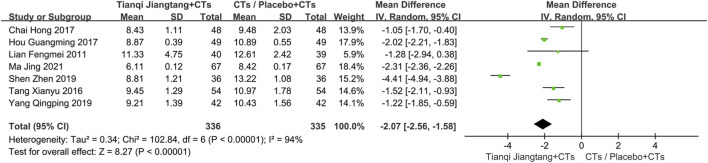
Overall pooled results forest plot comparing Tianqi Jiangtang Capsule plus conventional treatments (CTs) to placebo plus CTs or CTs alone on 2hPG.

Seven studies (Lian et al., 2011; Tang et al., 2016; Chai et al., 2017; Hou C., 2017; Shen, 2019; Yang et al., 2019; Ma et al., 2021) reported 2hPG levels (Figure 7), with significant heterogeneity among the studies (*P* < 0.00001, *I* ^*2*^ = 94%). A random-effects model was therefore employed for meta-analysis. The results indicated that the observation group had significantly lower 2hPG levels than the control group after treatment (*MD* = −2.07, 95% *CI* = −2.56 to −1.58, *P* < 0.00001) (Table 2).

**FIGURE 8:**
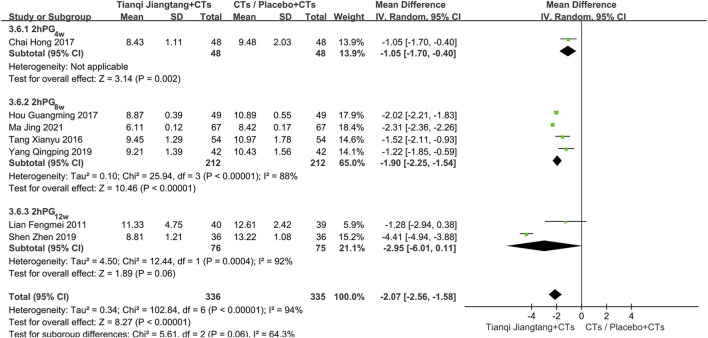
Subgroup analysis forest plot comparing Tianqi Jiangtang Capsule plus conventional treatments (CTs) to placebo plus CTs or CTs alone on 2hPG.

Subgroup analysis based on intervention duration was conducted. One study (Chai et al., 2017) with a 4 week intervention showed a significant difference between the two groups (*MD* = −1.05, 95% *CI* = −1.70 to −0.40, *P* = 0.002). Among the four studies (Tang et al., 2016; Hou C., 2017; Yang et al., 2019; Ma et al., 2021) with an 8 week intervention, significant heterogeneity was observed (*P* < 0.01, *I* ^*2*^ = 88%). After reviewing the full texts, variations in study populations and intervention protocols were identified as potential sources of heterogeneity. Using a random-effects model, the pooled effect size demonstrated significantly lower 2hPG levels in the observation group (*MD* = −1.90, 95% *CI* = −2.25 to −1.54, *P* < 0.00001). Two studies (Lian et al., 2011; Shen, 2019) with a 12 week intervention exhibited significant heterogeneity (*P* < 0.01, *I* ^*2*^ = 92%). A random-effects model was applied, and the meta-analysis results showed no significant difference between the two groups (*MD* = −2.95, 95% *CI* = −6.01 to 0.11, *P* = 0.06). Differences in the enrolled populations were considered a possible source of heterogeneity (Figure 8).

### 3.5 Secondary outcomes

#### 3.5.1 hs-CRP

Three studies (Cao et al., 2015; Chai et al., 2017; Wu et al., 2017) used hs-CRP as an outcome measure (Supplementary Figure S1). Sensitivity analysis indicated robust results, while significant heterogeneity was observed among the studies (*P* < 0.00001, *I* ^*2*^ = 91%). A random-effects model was therefore applied for meta-analysis. The results demonstrated that, compared with conventional therapy alone or combined with placebo, the addition of TJC led to a statistically significant improvement in hs-CRP levels (*MD* = −2.51, 95% *CI* = −3.71 to −1.30, *P* < 0.0001) (Table 2).

Subgroup analysis based on intervention duration showed that one study (Chai et al., 2017) with a 4 week treatment period reported a significant difference between the two groups (*MD* = −1.15, 95% *CI* = −1.62 to −0.68, *P* < 0.00001). Two studies (Cao et al., 2015; Wu et al., 2017) with an 8 week intervention showed low heterogeneity (*P* = 0.28, *I* ^*2*^ = 13%). The results indicated that the observation group was superior to the control group (*MD* = −3.08, 95% *CI* = −3.32 to −2.84, *P* < 0.00001) (Supplementary Figure S2).

#### 3.5.2 IL-6

Three studies (Wu et al., 2017; Xu et al., 2018; Yang et al., 2019) used IL-6 as an outcome measure (Supplementary Figure S3). Sensitivity analysis indicated robust results, with low heterogeneity observed among the studies (*P* = 0.93, *I* ^*2*^ = 0%). A fixed-effect model was therefore applied for meta-analysis. The results demonstrated a statistically significant reduction in IL-6 levels in the observation group compared to the control group (*MD* = −3.43, 95% *CI* = −3.87 to −2.98, *P* < 0.00001) (Table 2).

**FIGURE 9:**
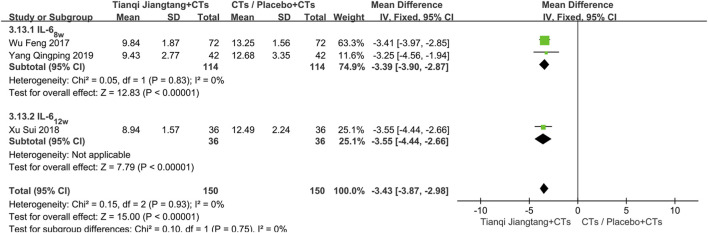
Subgroup analysis forest plot comparing Tianqi Jiangtang Capsule plus conventional treatments (CTs) to placebo plus CTs or CTs alone on IL-6.

Subgroup analysis was performed based on treatment duration. Two studies had a treatment duration of 8 weeks (Wu et al., 2017; Yang et al., 2019), showing no significant heterogeneity (*P* = 0.83, *I* ^*2*^ = 0%). The meta-analysis results indicated a statistically significant difference in efficacy between the two groups (*MD* = −3.55, 95% *CI* = −4.44 to −2.66, *P* < 0.00001). One study with a 12 week treatment duration (Xu et al., 2018) also demonstrated a significant difference between groups (*MD* = −3.08, 95% *CI* = −3.32 to −2.84, *P* < 0.00001) (Figure 9).

#### 3.5.3 TNF-α

Four studies (Cao et al., 2015; Wu et al., 2017; Xu et al., 2018; Yang et al., 2019) used TNF-α as an outcome measure (Supplementary Figure S4). Sensitivity analysis indicated robust results, with significant heterogeneity observed among the studies (*P* < 0.00001, *I* ^*2*^ = 97%). A random-effects model was therefore applied for meta-analysis. The results demonstrated that the addition of Tianqi Jiangtang Granule to conventional therapy led to a statistically significant improvement in TNF-α levels compared to conventional treatment alone or combined with placebo (*MD* = −7.66, 95% *CI* = −11.26 to −4.06, *P* < 0.0001) (Table 2).

Subgroup analysis based on intervention duration showed that three studies (Cao et al., 2015; Wu et al., 2017; Yang et al., 2019) with an 8 week intervention exhibited substantial heterogeneity (*P* < 0.01, *I* ^*2*^ = 97%). A random-effects model was used, and the results indicated a significant difference between the two groups (*MD* = −6.33, 95% *CI* = −9.90 to −2.75, *P* = 0.0005). After excluding the study by Wu F. (2017) (Wu et al., 2017), heterogeneity decreased significantly (*P* = 0.36, *I* ^*2*^ = 0%). A full-text review revealed that the study by Wu F. (2017) (Wu et al., 2017) involved patients with early-stage diabetic kidney disease, while the other two studies included general patients with diabetes, suggesting that differences in population characteristics may have contributed to the heterogeneity. One study (Xu et al., 2018) with a 12 week intervention showed superior efficacy in the observation group compared to the control group (*MD* = −12.11, 95% *CI* = −15.24 to −8.98, *P* < 0.00001) (Supplementary Figure S5).

#### 3.5.4 TC

Six studies (Lian et al., 2011; Tang et al., 2016; Chai et al., 2017; Hou C., 2017; Wu and Gao, 2017; Qiao, 2019) used TC as an outcome measure (Supplementary Figure S6). Sensitivity analysis indicated robust results, with significant heterogeneity observed among the studies (*P* < 0.00001, *I* ^*2*^ = 87%). A random-effects model was therefore employed for meta-analysis. The results demonstrated a statistically significant reduction in TC levels in the observation group compared to the control group (*MD* = −0.52, 95% *CI* = −0.70 to −0.35, *P* < 0.00001) (Table 2).

**FIGURE 10:**
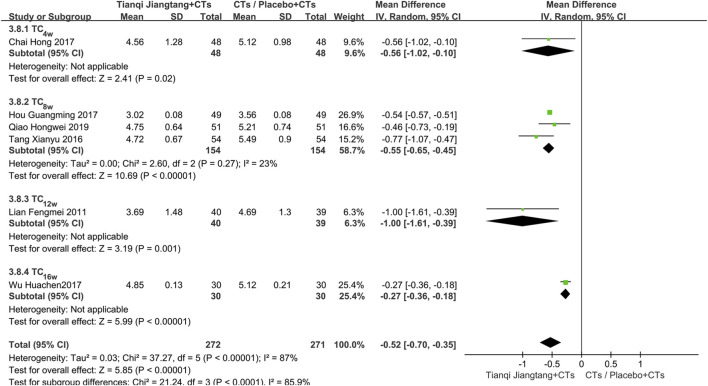
Subgroup analysis forest plot comparing Tianqi Jiangtang Capsule plus conventional treatments (CTs) to placebo plus CTs or CTs alone on TC.

Subgroup analysis based on intervention duration was performed. One study (Chai et al., 2017) with a 4 week intervention period showed a significant difference between the two groups (*MD* = −0.56, 95% *CI* = −1.02 to −0.10, P = 0.02). Three studies (Tang et al., 2016; Hou C., 2017; Qiao, 2019) with an 8 week intervention exhibited low heterogeneity (*P* = 0.27, *I* ^*2*^ = 23%). The pooled effect size indicated significantly lower TC levels in the observation group (*MD* = −0.55, 95% *CI* = −0.65 to −0.45, *P* < 0.00001). One study (Lian et al., 2011) with a 12 week intervention showed a significant difference between the groups (*MD* = −1.00, 95% *CI* = −1.61 to −0.39, *P* = 0.001). One study (Wu and Gao, 2017) with a 16 week intervention also demonstrated lower TC levels in the observation group (*MD* = −0.27, 95% *CI* = −0.36 to −0.18, *P* < 0.00001) (Figure 10).

#### 3.5.5 TG

Six studies (Lian et al., 2011; Tang et al., 2016; Chai et al., 2017; Hou C., 2017; Wu and Gao, 2017; Qiao, 2019) reported TG levels as an outcome measure (Supplementary Figure S7). Sensitivity analysis suggested robust findings, with significant heterogeneity detected among the studies (*P* = 0.001, *I* ^*2*^ = 76%). A random-effects model was therefore used for meta-analysis. The results indicated a statistically significant reduction in TG levels in the observation group compared to the control group (*MD* = −0.24, 95% *CI* = −0.34 to −0.15, *P* < 0.00001) (Table 2).

Subgroup analysis based on intervention duration showed that one study (Chai et al., 2017) with a 4 week intervention period reported significantly lower TG levels in the observation group after treatment (*MD* = −0.17, 95% *CI* = −0.29 to −0.05, P = 0.004). Three studies (Tang et al., 2016; Hou C., 2017; Qiao, 2019) with an 8 week intervention showed no significant heterogeneity (*P* = 0.83, *I* ^*2*^ = 0%). The pooled effect size demonstrated a significant difference between the two groups (*MD* = −0.32, 95% *CI* = −0.34 to −0.30, *P* < 0.00001). One study (Lian et al., 2011) with a 12 week intervention found no significant difference between the observation and control groups (*MD* = −0.19, 95% *CI* = −0.53 to 0.15, *P* = 0.27). Another study (Wu and Gao, 2017) with a 16 week intervention showed lower TG levels in the observation group, with a result approaching statistical significance (*MD* = −0.11, 95% *CI* = −0.22 to 0.00, *P* = 0.05) (Supplementary Figure S8).

#### 3.5.6 LDL-C

Five studies (Lian et al., 2011; Tang et al., 2016; Chai et al., 2017; Hou C., 2017; Wu and Gao, 2017) reported LDL-C levels (Supplementary Figure S9). Significant heterogeneity was observed among these studies (*P* < 0.00001, *I* ^*2*^ = 94%), and a random-effects model was therefore applied for meta-analysis. The results indicated that the observation group had significantly lower LDL-C levels than the control group after treatment (*MD* = −0.94, 95% *CI* = −1.18 to −0.71, *P* < 0.00001) (Table 2).

Subgroup analysis based on intervention duration showed that one study (Chai et al., 2017) with a 4 week intervention period demonstrated a significant difference between the two groups (*MD* = −1.09, 95% *CI* = −1.22 to −0.96, *P* < 0.00001). Two studies (Tang et al., 2016; Hou C., 2017) with an 8 week intervention exhibited significant heterogeneity (*P* < 0.01, *I* ^*2*^ = 97%). After comparing the studies, it was noted that one study involved patients with diabetic kidney disease, while the other included individuals with early-stage diabetic kidney disease—suggesting that differences in study populations may explain the heterogeneity. Using a random-effects model, the pooled effect size showed significantly lower LDL-C levels in the observation group (*MD* = −1.09, 95% *CI* = −1.78 to −0.41, *P* = 0.002). One study (Lian et al., 2011) with a 12 week intervention showed a statistically significant difference between the groups (*MD* = −0.80, 95% *CI* = −1.26 to −0.34, *P* < 0.01). Another study (Wu and Gao, 2017) with a 16 week intervention also demonstrated lower LDL-C levels in the observation group (*MD* = −0.63, 95% *CI* = −0.76 to −0.50, *P* < 0.00001) (Supplementary Figure S10).

#### 3.5.7 BUN

Seven studies (Tang et al., 2016; Hou G., 2017; Hou C., 2017; Wu et al., 2017; Xu et al., 2018; Qiao, 2019; Shen, 2019) reported BUN levels (Supplementary Figure S11), with significant heterogeneity among them (*P* < 0.00001, *I* ^*2*^ = 89%). Sensitivity analysis indicated robust results. A random-effects model was therefore employed for meta-analysis, which showed that the observation group had significantly lower BUN levels than the control group after treatment (*MD* = −0.96, 95% *CI* = −1.17 to −0.76, *P* < 0.00001) (Table 2).

**FIGURE 11:**
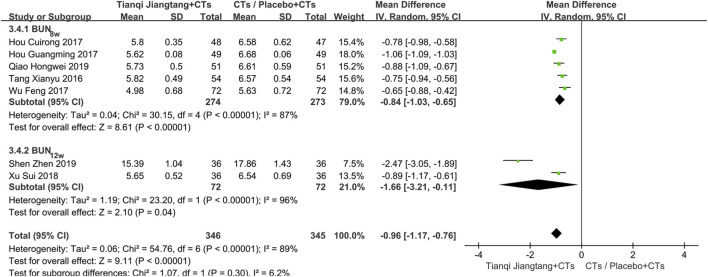
Subgroup analysis forest plot comparing Tianqi Jiangtang Capsule plus conventional treatments (CTs) to placebo plus CTs or CTs alone BUN.

Subgroup analysis based on intervention duration was performed. Among the five studies (Tang et al., 2016; Hou G., 2017; Hou C., 2017; Wu et al., 2017; Qiao, 2019) with an 8 week intervention period, significant heterogeneity was observed (*P* < 0.01, *I* ^*2*^ = 87%). After excluding the study Hou G. (2017) (Hou C., 2017), heterogeneity decreased markedly (*P* = 0.54, *I* ^*2*^ = 0%). A detailed review of the full texts suggested that differences in intervention protocols and study populations may have contributed to the heterogeneity. Using a random-effects model, the meta-analysis demonstrated a significant difference between the two groups (*MD* = −0.84, 95% *CI* = −1.03 to −0.65, *P* < 0.00001). Two studies (Xu et al., 2018; Shen, 2019) with a 12 week intervention exhibited significant heterogeneity (*P* < 0.01, *I* ^*2*^ = 96%). Comparative review revealed that one study enrolled patients with stage II–III diabetic kidney disease, while the other included participants with diabetic kidney disease without stage restrictions. Differences in population characteristics were identified as a potential source of heterogeneity. A random-effects model was applied, and the pooled effect size indicated significantly lower BUN levels in the observation group (*MD* = −1.66, 95% *CI* = −3.21 to −0.11, *P* = 0.04) (Figure 11).

#### 3.5.8 Scr

Seven studies (Tang et al., 2016; Hou G., 2017; Hou C., 2017; Wu et al., 2017; Xu et al., 2018; Qiao, 2019; Shen, 2019) reported Scr levels (Supplementary Figure S12), with significant heterogeneity observed among them (*P* < 0.00001, *I* ^*2*^ = 97%). Sensitivity analysis indicated that the results were robust. A random-effects model was therefore applied for meta-analysis, which showed significantly lower Scr levels in the observation group compared to the control group after treatment (*MD* = −18.53, 95% *CI* = −23.78 to −13.28, *P* < 0.00001) (Table 2).

Subgroup analysis based on intervention duration revealed that among the five studies (Tang et al., 2016; Hou G., 2017; Hou C., 2017; Wu et al., 2017; Qiao, 2019) with an 8 week intervention, heterogeneity was not significant (*P* = 0.36, *I* ^*2*^ = 8%). The analysis demonstrated a statistically significant difference between the two groups (*MD* = −10.39, 95% *CI* = −10.96 to −9.82, *P* < 0.00001). Two studies (Xu et al., 2018; Shen, 2019) with a 12 week intervention showed substantial heterogeneity (*P* < 0.01, *I* ^*2*^ = 99%). A comparative review indicated that differences in the enrolled populations may have contributed to the heterogeneity. Using a random-effects model, the pooled results indicated a non-significant reduction in Scr levels in the observation group (*MD* = −76.08, 95% *CI* = −185.01 to 32.85, P = 0.17) (Supplementary Figure S13).

### 3.6 Safety outcomes

Eight studies (Tang et al., 2016; Chai et al., 2017; Hou C., 2017; Wu et al., 2017; Xu et al., 2018; Qiao, 2019; Yang et al., 2019; Ma et al., 2021) reported on adverse events. Among these, six studies explicitly reported that no adverse drug reactions (ADRs) occurred. The remaining two studies (Xu et al., 2018; Yang et al., 2019) reported specific ADRs. One study (Xu et al., 2018) indicated that during the treatment period, one case of dizziness occurred in the control group, and one case of nausea and one case of gastric discomfort were reported in the treatment group. Another study (Yang et al., 2019) reported one case of hypoglycemia and one case of headache in the control group, and one case of abdominal pain, one case of vomiting, and one case of hypoglycemia in the treatment group. The remaining studies did not report any information regarding the occurrence of adverse drug reactions. Meta-analysis showed no statistically significant difference in the incidence of adverse reactions between the two groups (*RR* = 1.71, 95% *CI* = 0.39 to 7.44, *P* = 0.47). None of the studies reported any occurrence of serious adverse events (Table 2).

### 3.7 Sensitivity analysis

Sensitivity analysis was performed using the leave-one-out method. The results indicated that the significance of the outcomes remained unchanged regardless of which individual study was omitted. This suggests that the findings of this meta-analysis are robust.

### 3.8 Publication bias

**FIGURE 12:**
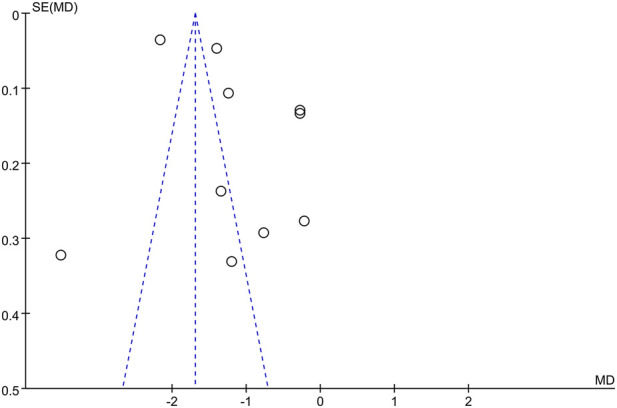
Funnel plot of HbA1c.

Since the number of studies was fewer than 10 for all other outcome measures, publication bias was evaluated only for HbA1c using a funnel plot. The results suggested the presence of potential publication bias (Figure 12). Further assessment using Egger’s and Begg’s tests confirmed significant statistical evidence (p < 0.001 and p = 0.002, respectively), suggesting potential small-study effects (Supplementary Figure S14). However, the trim-and-fill method did not detect any missing studies requiring imputation, and the adjusted effect size remained identical to the original estimate (*SMD* = −2.47, 95% *CI*: −4.46 to −0.49, Supplementary Figure S15). The adjusted forest plot is presented in Supplementary Figure S16, and the Egger’s regression plot is shown in Supplementary Figure S17.

### 3.9 Certainty assessment

The certainty of the evidence was rated using the GRADE method (Table 3). The overall certainty was low to very low. This was mainly due to a high risk of bias, imprecision and inconsistency.

**TABLE 3:** Certainty of evidence for Tianqi Jiangtang in the treatment of patients with diabetes mellitus.

Outcomes	No. of participants (studies)	Anticipated absolute effects (95% *CI*)		Certainty of the evidence
		Risk with CG	Risk difference with, E.G.,	
HbA1c	877 (10)	The mean HbA1c ranged from 6.87 to 10.31	The mean HbA1c in the, E.G., was 1.22 fewer (0.74 fewer to 1.70 fewer)	⊕〇〇〇 Very Low^a,d,e^
FPG	745 (8)	The mean FPG ranged from 6.27 to 9.61	The mean FPG in the, E.G., was 1.37 fewer (0.99 fewer to 1.74 fewer)	⊕⊕〇〇 Low^a,d^
2hPG	671 (7)	The mean 2hPG ranged from 8.42 to 13.22	The mean 2hPG in the, E.G., was 2.07 fewer (1.58 fewer to 2.56 fewer)	⊕⊕〇〇 Low^a,d^

Abbreviation: CTs, conventional treatments; E.G., experiment group; CG, control group; a, poor methodology, including the method of randomization and blinding; b, only one study provided data; c, small sample; size; d, I2≥ 50% for heterogeneity; e, publication bias.

## 4 Discussion

Our study primarily evaluated the glucose-lowering efficacy of Tianqi Jiangtang Capsule in diabetic patients. Additionally, we evaluated the capsule’s ability to suppress systemic inflammation, regulate lipid metabolism, and improve renal function.

Our findings demonstrate that TJC significantly reduces HbA1c, FPG, and 2hPG levels in diabetic patients. HbA1c serves as a reliable indicator of long-term glycemic status, being less affected by short-term physiological fluctuations (Wang et al., 2023). The marked reduction in HbA1c indicates TJC’s effectiveness in stabilizing glucose metabolism. Although no statistically significant improvements in FPG and 2hPG were observed in the 12 week subgroup (likely due to substantial heterogeneity and limited sample size), the observed downward trend still supports TJC’s regulatory effect on glucose metabolism. Furthermore, our results show that TJC substantially improves lipid metabolism, inflammatory markers, and renal function. These systemic benefits are interconnected through two key pathophysiological factors: insulin resistance and chronic inflammation.

Proteomic and metabolomic studies suggest that TJC exerts multi-targeted improvement in insulin resistance. In animal models following TJC intervention, alterations in key serum protein levels were observed, manifested as increased apolipoprotein E (ApoE), apolipoprotein A1 (ApoA1), and transthyretin (TTR) alongside decreased haptoglobin (Hp) and serum amyloid P-component (SAP) (Zhang et al., 2010). Additionally, upregulation was noted in insulin-independent pathways including glucose transporter type 4 (GluT-4) and mitogen-activated protein kinase (MAPK) signaling, as well as the lipid metabolism pathway mediated by upregulation of perixisome proliferator-activated receptor alpha (PPAR-α) (Zhang et al., 2009). Human metabolomic studies on adipose tissue indicate that TJC may participate in the restoration of metabolic processes involving phospholipids, glycolipids, nucleosides, and carnitines (Yu et al., 2011). In contrast to Western medications like metformin, which primarily act on Adenosine 5′-monophosphate (AMP)-activated protein kinase (AMPK) and GluT-4 signaling pathways (Herman et al., 2022), TJC exerts multiple therapeutic benefits by targeting a broader range of molecular pathways. These mechanisms collectively enhance glucose uptake and utilization, improve insulin resistance, and regulate lipid profiles.

Another crucial aspect of TJC’s mechanism lies in its impact on chronic inflammation. Previous studies have confirmed that systemic chronic inflammation serves as a key driver in the development and progression of diabetes and its complications. It can not only perpetuate insulin resistance but also directly contribute to end-organ damage (Yaribeygi et al., 2019). Elevated hs-CRP levels are closely associated with an increased risk of T2DM in middle-aged and elderly Chinese populations (Yang et al., 2021). Furthermore, longitudinal changes in hs-CRP show a significant association with all-cause mortality risk (Wang et al., 2024). IL-6 inhibits SLC39A5 expression, leading to hyperglucagonemia-associated hyperglycemia, which is closely linked to the development of diabetic kidney disease and diabetes-related cardiovascular complications (Kreiner et al., 2022; Chen et al., 2023). TNF-α may mediate inflammatory responses by activating transcription factors such as kappa B kinase beta (IKKβ), c-Jun N-terminal kinase (JNK), and nuclear factor kappa-B (NF-κB) (Akash et al., 2018). Its upregulation of TNF-related apoptosis-inducing ligand (TRAIL) and death receptor 5 (DR5) expression exacerbates the progression of diabetic nephropathy (Lv et al., 2025). By concurrently alleviating insulin resistance and inflammatory levels, TJC disrupts this vicious cycle between metabolic dysregulation and tissue injury, suggesting potential ameliorative effects against a spectrum of diabetic complications.

Our study reveals that TJC’s renoprotective effects further corroborate the aforementioned perspective (significant reductions in BUN and Scr levels). Animal experiments demonstrated that diabetic kidney disease (DKD) rats treated with high dose TJC exhibited significant reductions in blood glucose, lipid levels, proteinuria, and the pro-fibrotic factor serum transforming growth factor-β1 (TGF-β1), along with enhanced activities of antioxidant enzymes superoxide dismutase (SOD) and glutathione peroxidase (GSH-Px). Histopathological examination of renal tissue showed a decreased number of sclerotic glomeruli and alleviated edema in renal tubular epithelial cells (Chen et al., 2017). This confirms that TJC ameliorates the progression of diabetic kidney disease through its antioxidant and anti-fibrotic functions. Additional studies have reported that TJC can improve blood viscosity, inhibit platelet aggregation, and enhance cognitive function in elderly diabetic patients with cerebral microangiopathy, as well as improve peripheral vascular elasticity in early-stage diabetes (Cheng and Yin, 2017; Wu and Gao, 2017). These effects may represent downstream manifestations of its synergistic actions on glucose homeostasis, lipid metabolism, and inflammatory pathways.

In summary, the integrated evidence presented in this paper indicates that TJC contributes to the improvement of glucose homeostasis in diabetic patients. Its glucose-lowering effect is not an isolated action, but rather reflects its role as a multi-target therapeutic agent acting on an interconnected network of metabolic, inflammatory, and oxidative stress pathways. Beyond TJC, various other Chinese botanical drugs and proprietary Chinese patent medicines have demonstrated advantages in the long-term management of diabetes. For instance, Shenqi Jiangtang Capsule, which shares similarities with TJC, has been reported to reduce levels of inflammatory markers such as TNF-α, IL-1β, CRP, and IL-6 (Cheng et al., 2025). This effect may be associated with its modulation of pathways such as mitogen-activated protein kinases (MAPK) and AKT serine/threonine kinase (AKT) (Chen et al., 2025). This mechanism partially overlaps with that of TJC. Additionally, other preparations such as Qigui Didang Formula (Wang et al., 2022), Jinqi Jiangtang Capsule (Hao et al., 2025), and Wumei Pill (Huang et al., 2025) have been shown to confer additional benefits beyond glucose-lowering, including suppression of inflammation, improvement of renal function, and regulation of lipid metabolism. A recent systematic review evaluated the efficacy of 23 Chinese botanical drugs for type 2 diabetes. The results demonstrated that the combination of Chinese botanical drugs with conventional Western medications yielded superior therapeutic efficacy in improving insulin resistance and dyslipidemia compared to Western medications alone (Ni et al., 2025). The comprehensive mechanism of action of TJC and similar Chinese medicines effectively compensates for the limitations of conventional diabetes treatments, which include the single-target focus of oral hypoglycemic agents and insulin therapy, the potential risks of target organ damage, and other adverse effects. Therefore, we propose that an integrated therapeutic approach combining Chinese botanical drugs with conventional hypoglycemic agents represents a more holistic strategy for managing type 2 diabetes and mitigating its associated complications.

Due to the limited number of included studies, publication bias was assessed only for the HbA1c outcome. The funnel plot suggested the possible presence of publication bias. Subsequent Egger’s and Begg’s tests yielded statistically significant results. However, the trim-and-fill method did not identify any missing studies requiring imputation, and the adjusted effect size remained unchanged. This phenomenon may be attributed to the substantial heterogeneity among the included studies and their relatively small sample sizes. Although the adjusted results remained statistically significant, there remains a possibility that small-scale studies may overestimate the magnitude of the effect. Thus, the findings warrant further validation through larger-scale, high-quality studies. Sensitivity analysis showed that the significance of the overall results did not change substantially with the omission of any individual study. Therefore, we consider the study findings to be robust. None of the included studies reported the implementation of blinding or allocation concealment, which may introduce potential bias. Although the use of objective outcome measures thus reduces the likelihood of measurement bias, the presence of selection bias and performance bias may still have led to an overestimation of the intervention effects.

No significant adverse reactions were reported in most studies during the treatment period. Mild gastrointestinal symptoms such as nausea, vomiting, and abdominal pain were occasionally observed but did not require special intervention. However, five studies did not report the occurrence of ADRs, and none of the included studies established predefined indicators for AEs. Therefore, the safety profile of TJC requires further confirmation. Future studies should provide more detailed reporting on the side effects, drug interactions, and overall safety profile of TJC.

This study has several limitations. First, all included RCTs were of low to moderate quality, and the lack of large high-quality RCTs may introduce bias. Second, the study populations were exclusively Chinese, limiting generalizability to other ethnicities, climates, and regions. Additionally, most studies did not clearly describe blinding methods, reducing the quality of evidence. Future studies should emphasize allocation concealment and strict double-blind designs to enhance standardization and credibility. The studies included in this systematic review exhibited considerable heterogeneity. We only performed subgroup analyses based on treatment duration. Due to constraints including incomplete baseline data reporting, unclear population classifications, and heterogeneous control medications in the original studies, it was not feasible to form meaningful subgroups for other analyses such as those based on type of control therapy, disease subtype, baseline HbA1c level, duration of diabetes, or study quality. Consequently, the inability to fully account for the sources of heterogeneity represents a limitation of this review. Finally, the short treatment and follow-up durations in the included studies preclude conclusions regarding the long-term effects of TJC. Extended observation periods are recommended in future research.

## 5 Conclusion

Our meta-analysis mainly evaluated the glucose-lowering efficacy of TJC. We discovered its additional benefits in inhibiting inflammation responses, improving lipid metabolism and protecting renal function. These findings suggest that beyond glycemic control, TJC may possess broader clinical significance by ameliorating inflammatory markers in diabetic patients. This supports its potential application for diabetic complications, such as diabetic kidney disease. However, the safety profile and scope of application of TJC require further investigation to be substantiated.

## Data Availability

The original contributions presented in the study are included in the article/Supplementary Material, further inquiries can be directed to the corresponding authors.
