# Supplementary material for: Tianqi Jiangtang Capsule in the treatment of patients with diabetes: a systematic review and meta-analysis
Source: Front Pharmacol. 2026 Jan 21;16:1719112. doi: 10.3389/fphar.2025.1719112 (PMC12868117; doi:10.3389/fphar.2025.1719112)
Supplement: Supplementary file 1 [file Supplementaryfile1.docx]

Supplementary Material

# Supplementary Tables

## Table S1. The search strategies for all databases.

| **The search strategy for PubMed** | |
| --- | --- |
| **Number** | **Search terms** |
| #1 | Diabetes Mellitus[MeSH Terms] |
| #2 | (((((((((Diabetes Mellitus[Title/Abstract])) OR (Diabetes Insipidus[Title/Abstract])) OR (Diet, Diabetic[Title/Abstract])) OR (Gastroparesis[Title/Abstract])) OR (Glucose Intolerance[Title/Abstract])) OR (Glycation End Products, Advanced[Title/Abstract])) OR (Prediabetic State[Title/Abstract])) OR (Scleredema Adultorum[Title/Abstract])) AND (((((tianqijiangtang capsule[Title/Abstract]) OR (tianqi jiangtang capsule[Title/Abstract])) OR (tianqi jiangtang[Title/Abstract])) OR (tianqi[Title/Abstract])) OR (tianqi hypoglycemic capsule[Title/Abstract])) |
| #3 | #1 OR #2 |
| #4 | ((((tianqijiangtang capsule[Title/Abstract]) OR (tianqi jiangtang capsule[Title/Abstract])) OR (tianqi jiangtang[Title/Abstract])) OR (tianqi[Title/Abstract])) OR (tianqi hypoglycemic capsule[Title/Abstract]) |
| #5 | #3 AND #4 |
| **The search strategy for Embase** | |
| **Number** | **Search terms** |
| #1 | 'Diabetes Mellitus'/exp |
| #2 | 'Diabetes Insipidus*':ab,ti OR 'Diet, Diabetic*':ab,ti OR 'Gastroparesis*':ab,ti OR 'Glucose Intolerance*':ab,ti OR 'Glycation End Products, Advanced*':ab,ti OR 'Prediabetic State*':ab,ti OR 'Scleredema Adultorum*':ab,ti |
| #3 | #1 OR #2 |
| #4 | 'tianqijiangtang capsule':ab,ti OR 'tianqi jiangtang capsule':ab,ti OR 'tianqi jiangtang':ab,ti OR 'tianqi':ab,ti OR 'tianqi hypoglycemic capsule':ab,ti OR 'tianqijiangtang':ab,ti |
| #5 | #3 AND #4 |
| **The search strategy for Cochrane Library** | |
| **Number** | **Search terms** |
| #1 | MeSH descriptor: [Diabetes Mellitus] explode all trees |
| #2 | (Diabetes Mellitus):ti,ab,kw OR (Diabetes Insipidus):ti,ab,kw OR (Diet, Diabetic):ti,ab,kw OR (Gastroparesis):ti,ab,kw OR (Glucose Intolerance):ti,ab,kw OR (Glycation End Products, Advanced):ti,ab,kw OR (Prediabetic State):ti,ab,kw OR (Scleredema Adultorum):ti,ab,kw |
| #3 | #1 OR #2 |
| #4 | (tianqijiangtang capsule):ti,ab,kw OR (tianqi jiangtang capsule):ti,ab,kw OR (tianqi jiangtang):ti,ab,kw OR (tianqi):ti,ab,kw OR (tianqi hypoglycemic capsule):ti,ab,kw OR (tianqijiangtang):ti,ab,kw |
| #5 | #3 AND #4 |
| **The search strategy for ClinicalTrials.gov** | |
| **Number** | **Search terms** |
| #1 | Diabetes Mellitus OR Diabetes Insipidus OR Diet, Diabetic OR Gastroparesis OR Glucose Intolerance OR Glycation End Products, Advanced OR Prediabetic State OR Scleredema Adultorum |
| #2 | tianqijiangtang capsule OR tianqi jiangtang capsule OR tianqi jiangtang OR tianqi OR tianqi hypoglycemic capsule |
| #3 | #1 AND #2 |
| **The search strategy for Web of Science** | |
| **Number** | **Search terms** |
| #1 | ((Diabetes Mellitus) OR (Diabetes Insipidus) OR (Diet, Diabetic) OR (Gastroparesis) OR (Glucose Intolerance) OR (Glycation End Products, Advanced) OR (Prediabetic State) OR (Scleredema Adultorum)) |
| #2 | ((tianqijiangtang capsule) OR (tianqi jiangtang capsule) OR (tianqi jiangtang) OR (tianqi) OR (tianqi hypoglycemic capsule)) |
| #3 | #1 AND #2 |
| **The search strategy for CNKI** | |
| (SU = '天芪降糖胶囊' OR SU = '天芪降糖') AND (SU= '糖尿病') | |
| **The search strategy for WanFang** | |
| 主题:("天芪降糖胶囊" or "天芪降糖") and 主题:("糖尿病") | |
| **The search strategy for VIP** | |
| ((M=天芪降糖胶囊 OR 天芪降糖) OR (R=天芪降糖胶囊 OR 天芪降糖)) AND ((M=糖尿病) OR (R=糖尿病)) | |
| **The search strategy for CBM** | |
| #1 | "糖尿病"[常用字段:智能] |
| #2 | "天芪降糖胶囊"[常用字段:智能] OR "天芪降糖"[常用字段:智能] |
| #3 | #1 AND #2 |

**Table S2**. The list of excluded reports.

| **Report excluded** | **Reason** |
| --- | --- |
| Hu Hao 2017 | Lack of efficacy outcomes |
| Wu Huachen 2017 | Lack of efficacy outcomes |
| Yang Fengyu 2016 | Lack of efficacy outcomes |
| Cheng Yuan 2017 | Non random |

**References**

HAO H. (2017) Observation on the Therapeutic Effect of Tianqi Jiangtang Capsules in the Treatment of Early Diabetic Nephropathy [J]. China Health Care & Nutrition, 2017, 27(29): 155.

HUACHEN W, YINGCHUN H, JIE G, and LIPING L. Evaluation of Vascular Wall Elasticityoflower Extremity Arteryin T2DM Patients Treated with Tianqijiangtang Capsule by Ultrasound Elastography [J]. Journal of Aerospace Medicine, 2017, 28(10): 1175-8.

FENGYU Y, DONGXU L. (2016) Effect of Tianqi Jiangtang capsule on early diabetic nephropathy [J]. Journal of Aerospace Medicine, 2016, 27(4): 491-2.

YUAN C, ZI-JIE Y. (2017) Study on the effect of Tianqi Jiangtang capsules combined with metformin on blood viscosity,cognitive disorder in elderly patients with type 2 diabetes mellitus complicated with cerebral microvascular lesions [J]. Chinese Journal of Biochemical Pharmaceutics, 2017, 37(7).

**Table S3**. The summary table of the studies included.

| **Study** | **Formulation** | **Source** | **Species** | **Quality control reported?**  **(Y/N)** | **Batch number?**  **(Y/N)** | **Chemical analysis reported?**  **(Y/N)** | **A chemical characterisation of the preparation** |
| --- | --- | --- | --- | --- | --- | --- | --- |
| Ma Jing | Tianqi Jiangtang Capsules (Specification: 0.32 g/capsule) | Heilongjiang Weiming Tianren Pharmaceutical Co., Ltd | - *Astragalus membranaceus* Fisch. ex Bunge [Fabaceae; Astragali Radix], root and rhizome. - *Trichosanthes kirilowii* Maxim. [Cucurbitaceae; Trichosanthis Radix], root - *Ligustrum lucidum* W.T.Aiton [Oleaceae; Ligustri Lucidi Fructus], ripe fruit. - *Dendrobium nobile* Lindl. [Orchidaceae; Dendrobii Caulis], stem. - *Panax ginseng* C.A.Mey. [Araliaceae; Ginseng Radix et Rhizoma], root. - *Lycium chinense* Mill. [Solanaceae; Lycii Cortex], dried root bark. - *Coptis chinensis* Franch. [Ranumculaceae; Coptidis Rhizoma], rhizome. - *Cornus officinalis* Siebold & Zucc. [Cornaceae; Corni Fructus], ripe pulp. - *Eclipta prostrata (L.) L.* [Asteraceae; Ecliptae Herba], dried Above-Ground Part. - *Rhus chinensis* Mill. [Anacardiaceae; Galla Chinensis], Galls on the leaves. | N | Y-190602 | N | HPLC and GC   1. *Astragalus membranaceus* Fisch. ex Bunge [Fabaceae; Astragali Radix]:   [Sitosterol,Î’-Sitosterol](http://www.tcmip.cn/ETCM/index.php/Home/Index/cf_details.html?id=66),v[4-Hydroxycoumarin,Folinic Acid](http://www.tcmip.cn/ETCM/index.php/Home/Index/cf_details.html?id=231), [Cetylic Acid,Hexadecanoic Acid,Palmitic Acid](http://www.tcmip.cn/ETCM/index.php/Home/Index/cf_details.html?id=258), [Sucrose](http://www.tcmip.cn/ETCM/index.php/Home/Index/cf_details.html?id=329), [Choline](http://www.tcmip.cn/ETCM/index.php/Home/Index/cf_details.html?id=776), [3-Hydroxycoumarin, Folic Acid](http://www.tcmip.cn/ETCM/index.php/Home/Index/cf_details.html?id=1465), [Kumatakenin](http://www.tcmip.cn/ETCM/index.php/Home/Index/cf_details.html?id=2436), [Î’etaine](http://www.tcmip.cn/ETCM/index.php/Home/Index/cf_details.html?id=2652), [Medicarpin](http://www.tcmip.cn/ETCM/index.php/Home/Index/cf_details.html?id=3006), [Acetyl Astragaloside I](http://www.tcmip.cn/ETCM/index.php/Home/Index/cf_details.html?id=3127), [Astragaloside I](http://www.tcmip.cn/ETCM/index.php/Home/Index/cf_details.html?id=3128), [Astragaloside Ii](http://www.tcmip.cn/ETCM/index.php/Home/Index/cf_details.html?id=3129), [Astragaloside Iii](http://www.tcmip.cn/ETCM/index.php/Home/Index/cf_details.html?id=3130), [Astragaloside Iv](http://www.tcmip.cn/ETCM/index.php/Home/Index/cf_details.html?id=3131), [Astragaloside V](http://www.tcmip.cn/ETCM/index.php/Home/Index/cf_details.html?id=3132), [Astragaloside Vi](http://www.tcmip.cn/ETCM/index.php/Home/Index/cf_details.html?id=3133), [Astragaloside Vii](http://www.tcmip.cn/ETCM/index.php/Home/Index/cf_details.html?id=3134), [Astragaloside Viii](http://www.tcmip.cn/ETCM/index.php/Home/Index/cf_details.html?id=3135), [Astramembrannin I](http://www.tcmip.cn/ETCM/index.php/Home/Index/cf_details.html?id=3136), [Astramembrannin Ii](http://www.tcmip.cn/ETCM/index.php/Home/Index/cf_details.html?id=3137), [Cycloastragenol](http://www.tcmip.cn/ETCM/index.php/Home/Index/cf_details.html?id=3138), [9,10-Dimethoxy-Pterocarpane-3-O-Î’-D-Glucoside](http://www.tcmip.cn/ETCM/index.php/Home/Index/cf_details.html?id=3139), [Glucuronic Acid](http://www.tcmip.cn/ETCM/index.php/Home/Index/cf_details.html?id=3140), [2'-Hydroxy-3',4'-Dimethoxy-Isoflavane-7-O-Î’-D-Glucoside](http://www.tcmip.cn/ETCM/index.php/Home/Index/cf_details.html?id=3141), [Isoastragaloside I](http://www.tcmip.cn/ETCM/index.php/Home/Index/cf_details.html?id=3142), [Isoastragaloside Ii](http://www.tcmip.cn/ETCM/index.php/Home/Index/cf_details.html?id=3143), [Astrasieversianin Ix](http://www.tcmip.cn/ETCM/index.php/Home/Index/cf_details.html?id=3144) (lowering blood pressure, strengthening the heart, enhancing hematopoietic function, preventing myocardial ischemia, dilating peripheral blood vessels, enhancing hypoxia tolerance, protecting the liver, antioxidation, delaying aging, improving renal function, immune regulation, antiviral, anti-tumor, anti-mutation, anti-stress, promoting anabolism)   1. *Trichosanthes kirilowii* Maxim. [Cucurbitaceae; Trichosanthis Radix]:   Starch, saponins, trichosanin protein and various amino acids, such as trans acid, arginine, glutamic acid, γ -aminobutyric acid and sugars, etc (anti-fertility, hypoglycemic, immune regulation, anti-bacterial, anti-viral, anti-HIV, anti-tumor)   1. *Ligustrum lucidum* W.T.Aiton [Oleaceae; Ligustri Lucidi Fructus]:   [Oleanolic Acid](http://www.tcmip.cn/ETCM/index.php/Home/Index/cf_details.html?id=310), [3Î’-Acetoxyolean-12-En-28-Oic Acid](http://www.tcmip.cn/ETCM/index.php/Home/Index/cf_details.html?id=552), [Ursolic Acid](http://www.tcmip.cn/ETCM/index.php/Home/Index/cf_details.html?id=1244), [Rhodioloside,Salidroside](http://www.tcmip.cn/ETCM/index.php/Home/Index/cf_details.html?id=1751), [D-Mannitol](http://www.tcmip.cn/ETCM/index.php/Home/Index/cf_details.html?id=2076), [3-O-Acetylursolic Acid](http://www.tcmip.cn/ETCM/index.php/Home/Index/cf_details.html?id=4368), [10-Hydroxyligustroside](http://www.tcmip.cn/ETCM/index.php/Home/Index/cf_details.html?id=4369), [Isonuezhenide](http://www.tcmip.cn/ETCM/index.php/Home/Index/cf_details.html?id=4370), [(8E)-Ligustroside](http://www.tcmip.cn/ETCM/index.php/Home/Index/cf_details.html?id=4371), [Ligustrosidic Acid](http://www.tcmip.cn/ETCM/index.php/Home/Index/cf_details.html?id=4372), [Lucidumoside A](http://www.tcmip.cn/ETCM/index.php/Home/Index/cf_details.html?id=4373), [Lucidumoside B](http://www.tcmip.cn/ETCM/index.php/Home/Index/cf_details.html?id=4374), [Lucidumoside C](http://www.tcmip.cn/ETCM/index.php/Home/Index/cf_details.html?id=4375), [Lucidumoside D](http://www.tcmip.cn/ETCM/index.php/Home/Index/cf_details.html?id=4376), [Neonuezhenide](http://www.tcmip.cn/ETCM/index.php/Home/Index/cf_details.html?id=4377), [Nuezhengalaside](http://www.tcmip.cn/ETCM/index.php/Home/Index/cf_details.html?id=4378), [Nuezhenidic Acid](http://www.tcmip.cn/ETCM/index.php/Home/Index/cf_details.html?id=4379), [(8E)-NÃ¼zhenide](http://www.tcmip.cn/ETCM/index.php/Home/Index/cf_details.html?id=4380), [Oleoside Dimethyl Ester](http://www.tcmip.cn/ETCM/index.php/Home/Index/cf_details.html?id=4381), [Oleuropein](http://www.tcmip.cn/ETCM/index.php/Home/Index/cf_details.html?id=4382), [Oleuropeinic Acid](http://www.tcmip.cn/ETCM/index.php/Home/Index/cf_details.html?id=4383) (increase white blood cells, enhance hematopoietic function, lower blood lipids, prevent atherosclerosis, protect the liver, lower blood sugar, regulate immunity, be anti-inflammatory, anti-allergic, anti-histamine, anti-bacterial, anti-mutation, and anti-eye damage)   1. *Dendrobium nobile* Lindl. [Orchidaceae; Dendrobii Caulis]:   [Nodakenetin](http://www.tcmip.cn/ETCM/index.php/Home/Index/cf_details.html?id=2144), [Dendrine](http://www.tcmip.cn/ETCM/index.php/Home/Index/cf_details.html?id=5098), [Dendrolasin](http://www.tcmip.cn/ETCM/index.php/Home/Index/cf_details.html?id=5180), [Evodiamine](http://www.tcmip.cn/ETCM/index.php/Home/Index/cf_details.html?id=5345), [Flavanthrinin](http://www.tcmip.cn/ETCM/index.php/Home/Index/cf_details.html?id=6844), [24-Hydroxy-11-Deoxyglycyrrhetic Acid](http://www.tcmip.cn/ETCM/index.php/Home/Index/cf_details.html?id=7113), [Rutaecarpine](http://www.tcmip.cn/ETCM/index.php/Home/Index/cf_details.html?id=7114), [Dentatin](http://www.tcmip.cn/ETCM/index.php/Home/Index/cf_details.html?id=7115), [2,3,5-Trihydroxy-4,9-Dimethoxyphenanthrene](http://www.tcmip.cn/ETCM/index.php/Home/Index/cf_details.html?id=7116), [2,8-Dihydroxy-3,4,7-Trimethoxy-9,10-Dihydrophenanthrene](http://www.tcmip.cn/ETCM/index.php/Home/Index/cf_details.html?id=7117), [2,8-Dihydroxy-3,4,7-Trimethoxyphenanthrene](http://www.tcmip.cn/ETCM/index.php/Home/Index/cf_details.html?id=7118), [2-Hydroxy-4,7-Dimethoxy-9,10-Dihydrophenanthrene](http://www.tcmip.cn/ETCM/index.php/Home/Index/cf_details.html?id=7119), [3,4,8-Trimethoxyphenanthrene-2,5-Diol](http://www.tcmip.cn/ETCM/index.php/Home/Index/cf_details.html?id=7120), [3-Hydroxy-2,4,7-Trimethoxy-9,10-Dihydrophenanthrene](http://www.tcmip.cn/ETCM/index.php/Home/Index/cf_details.html?id=7121), [3-O-Methylgigantol](http://www.tcmip.cn/ETCM/index.php/Home/Index/cf_details.html?id=7122), [5,7-Dimethoxyphenanthrene-2,6-Diol](http://www.tcmip.cn/ETCM/index.php/Home/Index/cf_details.html?id=7123), [6-Hydroxydendroxine](http://www.tcmip.cn/ETCM/index.php/Home/Index/cf_details.html?id=7124), [Dendroxine](http://www.tcmip.cn/ETCM/index.php/Home/Index/cf_details.html?id=7125), [Ephemeranthol A](http://www.tcmip.cn/ETCM/index.php/Home/Index/cf_details.html?id=7126), [Epheneranthol C](http://www.tcmip.cn/ETCM/index.php/Home/Index/cf_details.html?id=7127), [Erianthridin](http://www.tcmip.cn/ETCM/index.php/Home/Index/cf_details.html?id=7128), [Hircinol](http://www.tcmip.cn/ETCM/index.php/Home/Index/cf_details.html?id=7129), [Lusianthridin](http://www.tcmip.cn/ETCM/index.php/Home/Index/cf_details.html?id=7130), [Methyl 3-Carboxy-2Carboxymethyl-2-Hydroxypropanoate](http://www.tcmip.cn/ETCM/index.php/Home/Index/cf_details.html?id=7131), [Moscatilin](http://www.tcmip.cn/ETCM/index.php/Home/Index/cf_details.html?id=7132), [N-Methyldendrobium](http://www.tcmip.cn/ETCM/index.php/Home/Index/cf_details.html?id=7133), [Nobilonine](http://www.tcmip.cn/ETCM/index.php/Home/Index/cf_details.html?id=7134), [Nootkatone](http://www.tcmip.cn/ETCM/index.php/Home/Index/cf_details.html?id=7135) (lower blood pressure, inhibit smooth muscle, heal, delay aging, stimulate the uterus, regulate the immune system, and resist eye damage)   1. *Panax ginseng* C.A.Mey. [Araliaceae; Ginseng Radix et Rhizoma]:   [Alpha-Humulene,Humulene,Î‘-Humulene](http://www.tcmip.cn/ETCM/index.php/Home/Index/cf_details.html?id=38), [Î‘-Guriunene](http://www.tcmip.cn/ETCM/index.php/Home/Index/cf_details.html?id=52), [Sitosterol,Î’-Sitosterol](http://www.tcmip.cn/ETCM/index.php/Home/Index/cf_details.html?id=66), [(S)-1-Methyl-4-(6-Methylhepta-1,5-Dien-2-Yl)Cyclohex-1-Ene,Î’-Bisabolene](http://www.tcmip.cn/ETCM/index.php/Home/Index/cf_details.html?id=70), [(1S,2S)-2-Isopropenyl-4-Isopropylidene-1-Methyl-1-Vinylcyclohexane,Î“-Elemene](http://www.tcmip.cn/ETCM/index.php/Home/Index/cf_details.html?id=74), [Cis-9,Cis-12-Linoleic Acid,Inositol,Linoleic,Linoleic Acid](http://www.tcmip.cn/ETCM/index.php/Home/Index/cf_details.html?id=126), [(1R,9S)-4,11,11-Trimethyl-8-Methylenebicyclo[7.2.0]Undec-4-Ene,Caryophellene,Î’-Caryophyllene](http://www.tcmip.cn/ETCM/index.php/Home/Index/cf_details.html?id=158), [Stigmasterol](http://www.tcmip.cn/ETCM/index.php/Home/Index/cf_details.html?id=185), [Kaempferol](http://www.tcmip.cn/ETCM/index.php/Home/Index/cf_details.html?id=207), [Trifolin](http://www.tcmip.cn/ETCM/index.php/Home/Index/cf_details.html?id=208), [Cedrol,Eudesmol,Î‘-Cedrol](http://www.tcmip.cn/ETCM/index.php/Home/Index/cf_details.html?id=215), [3,4-Dihydroxybenzaldehyde,Hydroxybenzoic Acid,M-Hydroxybenzoic Acid,P-Hydroxybenzoic Acid,Salicylic Acid](http://www.tcmip.cn/ETCM/index.php/Home/Index/cf_details.html?id=218), [Cetylic Acid,Hexadecanoic Acid,Palmitic Acid](http://www.tcmip.cn/ETCM/index.php/Home/Index/cf_details.html?id=258), [Sucrose](http://www.tcmip.cn/ETCM/index.php/Home/Index/cf_details.html?id=329), [Succinic Acid](http://www.tcmip.cn/ETCM/index.php/Home/Index/cf_details.html?id=330), [Alexandrin,Daucosterol,Caproic Acid,Eleutheroside A,Sitogluside,Strumaroside,Î’-Sitosterol-Î’-D-Glucoside](http://www.tcmip.cn/ETCM/index.php/Home/Index/cf_details.html?id=331), [Riboflavine](http://www.tcmip.cn/ETCM/index.php/Home/Index/cf_details.html?id=370), [Xylose](http://www.tcmip.cn/ETCM/index.php/Home/Index/cf_details.html?id=440), [Citric Acid](http://www.tcmip.cn/ETCM/index.php/Home/Index/cf_details.html?id=446), [Î’-Humulene](http://www.tcmip.cn/ETCM/index.php/Home/Index/cf_details.html?id=534), [Campesterol,M-Cresol](http://www.tcmip.cn/ETCM/index.php/Home/Index/cf_details.html?id=585), [Adenosine,Adenine Nucleoside](http://www.tcmip.cn/ETCM/index.php/Home/Index/cf_details.html?id=615), [1-Heptadecanol](http://www.tcmip.cn/ETCM/index.php/Home/Index/cf_details.html?id=633), [Malic Acid](http://www.tcmip.cn/ETCM/index.php/Home/Index/cf_details.html?id=668), [Choline](http://www.tcmip.cn/ETCM/index.php/Home/Index/cf_details.html?id=776), [Hexadecane](http://www.tcmip.cn/ETCM/index.php/Home/Index/cf_details.html?id=902), [Beta-Elemene,Î’-Elemene](http://www.tcmip.cn/ETCM/index.php/Home/Index/cf_details.html?id=991), [Î’-Selinene](http://www.tcmip.cn/ETCM/index.php/Home/Index/cf_details.html?id=1044), [Î‘-Guaiene](http://www.tcmip.cn/ETCM/index.php/Home/Index/cf_details.html?id=1062), [Î’-Maaliene](http://www.tcmip.cn/ETCM/index.php/Home/Index/cf_details.html?id=1066), [N-Tridecane](http://www.tcmip.cn/ETCM/index.php/Home/Index/cf_details.html?id=1162), [Raffinose](http://www.tcmip.cn/ETCM/index.php/Home/Index/cf_details.html?id=1265), [Niacin,Nicotinic Acid](http://www.tcmip.cn/ETCM/index.php/Home/Index/cf_details.html?id=1382), [3-Hydroxycoumarin,Folic Acid](http://www.tcmip.cn/ETCM/index.php/Home/Index/cf_details.html?id=1465), [Tartaric Acid](http://www.tcmip.cn/ETCM/index.php/Home/Index/cf_details.html?id=1802), [Vitamin B1](http://www.tcmip.cn/ETCM/index.php/Home/Index/cf_details.html?id=1803), [Nonacosane](http://www.tcmip.cn/ETCM/index.php/Home/Index/cf_details.html?id=1811), [Vitamin B12](http://www.tcmip.cn/ETCM/index.php/Home/Index/cf_details.html?id=1934), [Fructose](http://www.tcmip.cn/ETCM/index.php/Home/Index/cf_details.html?id=1945), [Heptadecane](http://www.tcmip.cn/ETCM/index.php/Home/Index/cf_details.html?id=1949), [Mannose](http://www.tcmip.cn/ETCM/index.php/Home/Index/cf_details.html?id=1952), [Pentadecane](http://www.tcmip.cn/ETCM/index.php/Home/Index/cf_details.html?id=1963), [Rhamnose](http://www.tcmip.cn/ETCM/index.php/Home/Index/cf_details.html?id=1965), [Eicosane](http://www.tcmip.cn/ETCM/index.php/Home/Index/cf_details.html?id=2071), [Octanal](http://www.tcmip.cn/ETCM/index.php/Home/Index/cf_details.html?id=2222), [(1Ar,7R,7Ar,7Bs)-1,1,7,7A-Tetramethyl-1A,2,3,5,6,7,7A,7B-Octahydro-1H-Cyclopropa[A]Naphthalene,Calarene](http://www.tcmip.cn/ETCM/index.php/Home/Index/cf_details.html?id=2458), [Î’-Patchoulene](http://www.tcmip.cn/ETCM/index.php/Home/Index/cf_details.html?id=2483), [2,5-Dimethyl-7-Hydroxy Chromone](http://www.tcmip.cn/ETCM/index.php/Home/Index/cf_details.html?id=3028), [Glucuronic Acid](http://www.tcmip.cn/ETCM/index.php/Home/Index/cf_details.html?id=3140), [Dodecane](http://www.tcmip.cn/ETCM/index.php/Home/Index/cf_details.html?id=3758), [Mannose-B](http://www.tcmip.cn/ETCM/index.php/Home/Index/cf_details.html?id=3941), [Adenosine Triphosphate](http://www.tcmip.cn/ETCM/index.php/Home/Index/cf_details.html?id=3942), [Î‘-Cadinene](http://www.tcmip.cn/ETCM/index.php/Home/Index/cf_details.html?id=4414), [Î’-Farnesene](http://www.tcmip.cn/ETCM/index.php/Home/Index/cf_details.html?id=4421), [Alloaromadendrene](http://www.tcmip.cn/ETCM/index.php/Home/Index/cf_details.html?id=4606), [Aposcopolamine](http://www.tcmip.cn/ETCM/index.php/Home/Index/cf_details.html?id=4607), [Araloside A](http://www.tcmip.cn/ETCM/index.php/Home/Index/cf_details.html?id=4608), [Bicyclogermacrene](http://www.tcmip.cn/ETCM/index.php/Home/Index/cf_details.html?id=4609), [Biotin](http://www.tcmip.cn/ETCM/index.php/Home/Index/cf_details.html?id=4610), [Î“-Cadinene](http://www.tcmip.cn/ETCM/index.php/Home/Index/cf_details.html?id=4611), [Î”-Cadinol,Î”-Cadinene](http://www.tcmip.cn/ETCM/index.php/Home/Index/cf_details.html?id=4612), [Chikusetsusaponin Iii](http://www.tcmip.cn/ETCM/index.php/Home/Index/cf_details.html?id=4613), [Chikusetsusaponin Iv](http://www.tcmip.cn/ETCM/index.php/Home/Index/cf_details.html?id=4614), [Chloropanaxydiol](http://www.tcmip.cn/ETCM/index.php/Home/Index/cf_details.html?id=4615), [Deoxygomisin A](http://www.tcmip.cn/ETCM/index.php/Home/Index/cf_details.html?id=4616), [Dianthoside](http://www.tcmip.cn/ETCM/index.php/Home/Index/cf_details.html?id=4617), [Dibutyl Oxalate](http://www.tcmip.cn/ETCM/index.php/Home/Index/cf_details.html?id=4618), [2,6-Ditertbutyl-4-Methyl Phenol](http://www.tcmip.cn/ETCM/index.php/Home/Index/cf_details.html?id=4619), [Î”-Elemene](http://www.tcmip.cn/ETCM/index.php/Home/Index/cf_details.html?id=4620), [(9R,10S)-Epoxyheptadecan-4,6-Diyn-3-One](http://www.tcmip.cn/ETCM/index.php/Home/Index/cf_details.html?id=4621), [Ginsenol](http://www.tcmip.cn/ETCM/index.php/Home/Index/cf_details.html?id=4622), [Ginsenoside F1](http://www.tcmip.cn/ETCM/index.php/Home/Index/cf_details.html?id=4623), [Ginsenoside F4](http://www.tcmip.cn/ETCM/index.php/Home/Index/cf_details.html?id=4624), [Ginsenoside I](http://www.tcmip.cn/ETCM/index.php/Home/Index/cf_details.html?id=4625), [Ginsenoside Ia](http://www.tcmip.cn/ETCM/index.php/Home/Index/cf_details.html?id=4626), [Ginsenoside Ib](http://www.tcmip.cn/ETCM/index.php/Home/Index/cf_details.html?id=4627), [Ginsenoside Ic](http://www.tcmip.cn/ETCM/index.php/Home/Index/cf_details.html?id=4628), [Ginsenoside Ii](http://www.tcmip.cn/ETCM/index.php/Home/Index/cf_details.html?id=4629), [Ginsenoside Iii](http://www.tcmip.cn/ETCM/index.php/Home/Index/cf_details.html?id=4630), [Ginsenoside La](http://www.tcmip.cn/ETCM/index.php/Home/Index/cf_details.html?id=4631), [Ginsenoside Ra0](http://www.tcmip.cn/ETCM/index.php/Home/Index/cf_details.html?id=4632), [Ginsenoside Ra1](http://www.tcmip.cn/ETCM/index.php/Home/Index/cf_details.html?id=4633), [Ginsenoside Ra2](http://www.tcmip.cn/ETCM/index.php/Home/Index/cf_details.html?id=4634), [Ginsenoside Ra3](http://www.tcmip.cn/ETCM/index.php/Home/Index/cf_details.html?id=4635), [Ginsenoside Rb1](http://www.tcmip.cn/ETCM/index.php/Home/Index/cf_details.html?id=4636), [Ginsenoside Rb2](http://www.tcmip.cn/ETCM/index.php/Home/Index/cf_details.html?id=4637), [Ginsenoside Rb3](http://www.tcmip.cn/ETCM/index.php/Home/Index/cf_details.html?id=4638), [Ginsenoside Rc](http://www.tcmip.cn/ETCM/index.php/Home/Index/cf_details.html?id=4639), [Ginsenoside Rd](http://www.tcmip.cn/ETCM/index.php/Home/Index/cf_details.html?id=4640), [Ginsenoside Re](http://www.tcmip.cn/ETCM/index.php/Home/Index/cf_details.html?id=4641), [Ginsenoside Rf](http://www.tcmip.cn/ETCM/index.php/Home/Index/cf_details.html?id=4642), [Ginsenoside Rg1](http://www.tcmip.cn/ETCM/index.php/Home/Index/cf_details.html?id=4643), [Ginsenoside Rg2](http://www.tcmip.cn/ETCM/index.php/Home/Index/cf_details.html?id=4644), [20(S)-Ginsenoside Rg3](http://www.tcmip.cn/ETCM/index.php/Home/Index/cf_details.html?id=4645), [20(R)-Ginsenoside Rh1](http://www.tcmip.cn/ETCM/index.php/Home/Index/cf_details.html?id=4646), [Ginsenoside-Rh1](http://www.tcmip.cn/ETCM/index.php/Home/Index/cf_details.html?id=4647), [20(R)-Ginsenoside-Rh2](http://www.tcmip.cn/ETCM/index.php/Home/Index/cf_details.html?id=4648), [Ginsenoside Rh2](http://www.tcmip.cn/ETCM/index.php/Home/Index/cf_details.html?id=4649), [Ginsenoside Rh3](http://www.tcmip.cn/ETCM/index.php/Home/Index/cf_details.html?id=4650), [Ginsenoside Rh4](http://www.tcmip.cn/ETCM/index.php/Home/Index/cf_details.html?id=4651), [Ginsenoside R0](http://www.tcmip.cn/ETCM/index.php/Home/Index/cf_details.html?id=4652), [Ginsenoside Rs1](http://www.tcmip.cn/ETCM/index.php/Home/Index/cf_details.html?id=4653), [Ginsenoside Rs2](http://www.tcmip.cn/ETCM/index.php/Home/Index/cf_details.html?id=4654), [Ginsenoyne A](http://www.tcmip.cn/ETCM/index.php/Home/Index/cf_details.html?id=4655), [Ginsenoyne B](http://www.tcmip.cn/ETCM/index.php/Home/Index/cf_details.html?id=4656), [Ginsenoyne C](http://www.tcmip.cn/ETCM/index.php/Home/Index/cf_details.html?id=4657), [Ginsenoyne D](http://www.tcmip.cn/ETCM/index.php/Home/Index/cf_details.html?id=4658), [Ginsenoyne E](http://www.tcmip.cn/ETCM/index.php/Home/Index/cf_details.html?id=4659), [Ginsinsene](http://www.tcmip.cn/ETCM/index.php/Home/Index/cf_details.html?id=4660), [20-Glucosylginsenoside Rf](http://www.tcmip.cn/ETCM/index.php/Home/Index/cf_details.html?id=4661), [Gomisin A](http://www.tcmip.cn/ETCM/index.php/Home/Index/cf_details.html?id=4662), [Isocitric Acid](http://www.tcmip.cn/ETCM/index.php/Home/Index/cf_details.html?id=4663), [Isocitric Acid B](http://www.tcmip.cn/ETCM/index.php/Home/Index/cf_details.html?id=4664), [Isocitric Acid C](http://www.tcmip.cn/ETCM/index.php/Home/Index/cf_details.html?id=4665), [Isocitric Acid D](http://www.tcmip.cn/ETCM/index.php/Home/Index/cf_details.html?id=4666), [Malonylginsenoside Rb2](http://www.tcmip.cn/ETCM/index.php/Home/Index/cf_details.html?id=4667), [Malonylginsenoside Rc](http://www.tcmip.cn/ETCM/index.php/Home/Index/cf_details.html?id=4668), [6'-Malonylginsenoside Rd1](http://www.tcmip.cn/ETCM/index.php/Home/Index/cf_details.html?id=4669), [Maltose](http://www.tcmip.cn/ETCM/index.php/Home/Index/cf_details.html?id=4670), [1-Methoxy-(9R,10S)-Epoxyheptadecan-4,6-Diyn-3-One](http://www.tcmip.cn/ETCM/index.php/Home/Index/cf_details.html?id=4671), [Î‘-Muurolene](http://www.tcmip.cn/ETCM/index.php/Home/Index/cf_details.html?id=4672), [Î‘-Neoclovene](http://www.tcmip.cn/ETCM/index.php/Home/Index/cf_details.html?id=4673), [Î’-Neoclovene](http://www.tcmip.cn/ETCM/index.php/Home/Index/cf_details.html?id=4674), [Neointermedeol](http://www.tcmip.cn/ETCM/index.php/Home/Index/cf_details.html?id=4675), [Notoginsenoside R1](http://www.tcmip.cn/ETCM/index.php/Home/Index/cf_details.html?id=4676), [Notoginsenoside R4](http://www.tcmip.cn/ETCM/index.php/Home/Index/cf_details.html?id=4677), [Palmitin](http://www.tcmip.cn/ETCM/index.php/Home/Index/cf_details.html?id=4678), [Panacon](http://www.tcmip.cn/ETCM/index.php/Home/Index/cf_details.html?id=4679), [Panaginsene](http://www.tcmip.cn/ETCM/index.php/Home/Index/cf_details.html?id=4680), [Panasinsanol A](http://www.tcmip.cn/ETCM/index.php/Home/Index/cf_details.html?id=4681), [Panasinsanol B](http://www.tcmip.cn/ETCM/index.php/Home/Index/cf_details.html?id=4682), [Î’-Panasinsene](http://www.tcmip.cn/ETCM/index.php/Home/Index/cf_details.html?id=4683), [Î‘-Panasinsene](http://www.tcmip.cn/ETCM/index.php/Home/Index/cf_details.html?id=4684), [Panaxacol](http://www.tcmip.cn/ETCM/index.php/Home/Index/cf_details.html?id=4685), [Panaxadiol](http://www.tcmip.cn/ETCM/index.php/Home/Index/cf_details.html?id=4686), [Panaxatriol](http://www.tcmip.cn/ETCM/index.php/Home/Index/cf_details.html?id=4687), [Panaxene](http://www.tcmip.cn/ETCM/index.php/Home/Index/cf_details.html?id=4688), [Panaxydol](http://www.tcmip.cn/ETCM/index.php/Home/Index/cf_details.html?id=4689), [Panaxytriol](http://www.tcmip.cn/ETCM/index.php/Home/Index/cf_details.html?id=4690), [Protopanaxadiol](http://www.tcmip.cn/ETCM/index.php/Home/Index/cf_details.html?id=4691), [Protopanaxatriol](http://www.tcmip.cn/ETCM/index.php/Home/Index/cf_details.html?id=4692), [Pseudoginsenoside F11](http://www.tcmip.cn/ETCM/index.php/Home/Index/cf_details.html?id=4693), P[yruvic Acid](http://www.tcmip.cn/ETCM/index.php/Home/Index/cf_details.html?id=4694), [Quinquenoside R1](http://www.tcmip.cn/ETCM/index.php/Home/Index/cf_details.html?id=4695), [Î‘-Santalol](http://www.tcmip.cn/ETCM/index.php/Home/Index/cf_details.html?id=4696), [Selina-4(15),7(11)-Diene](http://www.tcmip.cn/ETCM/index.php/Home/Index/cf_details.html?id=4697), [Î‘-Selinene](http://www.tcmip.cn/ETCM/index.php/Home/Index/cf_details.html?id=4698), [Î“-Selinene](http://www.tcmip.cn/ETCM/index.php/Home/Index/cf_details.html?id=4699), [Î’-Sitosterol-3-(6-Linoleoyl)Glucopyranoside](http://www.tcmip.cn/ETCM/index.php/Home/Index/cf_details.html?id=4700), [Î’-Sitosterol-3-(6-Palmitoleoyl)Glucopyranoside](http://www.tcmip.cn/ETCM/index.php/Home/Index/cf_details.html?id=4701), [Î’-Sitosterol-3-(6-Stearoyl)Glucopyranoside](http://www.tcmip.cn/ETCM/index.php/Home/Index/cf_details.html?id=4702), [Stigmasterol-3-(6-Linoleoyl)Glucopyranoside](http://www.tcmip.cn/ETCM/index.php/Home/Index/cf_details.html?id=4703), [Stigmasterol-3-(6-Oleoyl)Glucopyranoside](http://www.tcmip.cn/ETCM/index.php/Home/Index/cf_details.html?id=4704), [Stigmasterol-3-(6-Stearoyl)Glucopyranoside](http://www.tcmip.cn/ETCM/index.php/Home/Index/cf_details.html?id=4705), [Stigmast-3-O-Î’-D-Glucopyanosyl-6-Hexadecanoate](http://www.tcmip.cn/ETCM/index.php/Home/Index/cf_details.html?id=4706), [Tetradecane](http://www.tcmip.cn/ETCM/index.php/Home/Index/cf_details.html?id=4707), [Widdrol](http://www.tcmip.cn/ETCM/index.php/Home/Index/cf_details.html?id=4708) (sedative and hypnotic, calming, anticonvulsant, antipyretic and cooling, intellectual, central nervous system excitation, striated muscle relaxation, blood pressure reduction, heart strengthening, anti-platelet aggregation, anti-myocardial ischemia, peripheral vascular dilation, enhanced hypoxia tolerance, anti-shock, lipid-lowering, anti-atherosclerosis, anti-gastric injury and anti-ulcer, anti-oxidation, anti-aging, anti-diuresis, thyroid function influence, and adrenal cortical function enhancement It can lower blood sugar, regulate the immune system, enhance the function of mononuclear macrophages, have adaptogenic effects, resist bacteria, tumors, hemolysis, stress, radiation, and promote anabolism)  6.*Lycium chinense* Mill. [Solanaceae; Lycii Cortex]:  [Pavilion,Scopoletin,Scopoletol,Trigonelline](http://www.tcmip.cn/ETCM/index.php/Home/Index/cf_details.html?id=84) , [Cis-9,Cis-12-Linoleic Acid,Inositol,Linoleic,Linoleic Acid](http://www.tcmip.cn/ETCM/index.php/Home/Index/cf_details.html?id=126), [Stigmasterol](http://www.tcmip.cn/ETCM/index.php/Home/Index/cf_details.html?id=185), [Cinnamic Acid](http://www.tcmip.cn/ETCM/index.php/Home/Index/cf_details.html?id=206), [Alexandrin,Daucosterol,Caproic Acid,Eleutheroside A,Sitogluside,Strumaroside,Î’-Sitosterol-Î’-D-Glucoside](http://www.tcmip.cn/ETCM/index.php/Home/Index/cf_details.html?id=331), [Campesterol](http://www.tcmip.cn/ETCM/index.php/Home/Index/cf_details.html?id=447), [Cholesterol](http://www.tcmip.cn/ETCM/index.php/Home/Index/cf_details.html?id=1189), [Triatricontane](http://www.tcmip.cn/ETCM/index.php/Home/Index/cf_details.html?id=1501), [Aurantiamide Acetate](http://www.tcmip.cn/ETCM/index.php/Home/Index/cf_details.html?id=2200), [N-Tricosane](http://www.tcmip.cn/ETCM/index.php/Home/Index/cf_details.html?id=2414), [Kukoamine A](http://www.tcmip.cn/ETCM/index.php/Home/Index/cf_details.html?id=2681), [Lyciumamide](http://www.tcmip.cn/ETCM/index.php/Home/Index/cf_details.html?id=6513), [Myricyl Acid,Melissic Acid,Triacontanoic Acid](http://www.tcmip.cn/ETCM/index.php/Home/Index/cf_details.html?id=6514), [Hyoscyamine](http://www.tcmip.cn/ETCM/index.php/Home/Index/cf_details.html?id=6515), [Atropine](http://www.tcmip.cn/ETCM/index.php/Home/Index/cf_details.html?id=6516), [Lyciumin A](http://www.tcmip.cn/ETCM/index.php/Home/Index/cf_details.html?id=6517), [Sugiol](http://www.tcmip.cn/ETCM/index.php/Home/Index/cf_details.html?id=6518), [Betaine](http://www.tcmip.cn/ETCM/index.php/Home/Index/cf_details.html?id=6519), [Î‘-Dimorphecolic Acid,9-Hydroxy-10E,12Z-Octadecadienoic Acid](http://www.tcmip.cn/ETCM/index.php/Home/Index/cf_details.html?id=6520), [Lyciumin B](http://www.tcmip.cn/ETCM/index.php/Home/Index/cf_details.html?id=6521), [9-Hotre,(9S,10E,12Z,15Z)-9-Hydroxy-10,12,15-Octadecatrienoic Acid](http://www.tcmip.cn/ETCM/index.php/Home/Index/cf_details.html?id=6522) (reduce fever, lower body temperature, lower blood pressure, increase white blood cells, lower blood lipids, stimulate the uterus, lower blood sugar, and has antibacterial and antiviral effects)  7.*Coptis chinensis* Franch. [Ranumculaceae; Coptidis Rhizoma]:  [3-O-trans ferulylquinic acid](http://www.tcmip.cn/ETCM/index.php/Home/Index/cf_details.html?id=17), [Berberine](http://www.tcmip.cn/ETCM/index.php/Home/Index/cf_details.html?id=465), [Coptisine](http://www.tcmip.cn/ETCM/index.php/Home/Index/cf_details.html?id=472), [Obaculactone](http://www.tcmip.cn/ETCM/index.php/Home/Index/cf_details.html?id=603), [Magnoflorine](http://www.tcmip.cn/ETCM/index.php/Home/Index/cf_details.html?id=822), [Limonin](http://www.tcmip.cn/ETCM/index.php/Home/Index/cf_details.html?id=1043), [Jatrorrhizine](http://www.tcmip.cn/ETCM/index.php/Home/Index/cf_details.html?id=2233), [Palmatine](http://www.tcmip.cn/ETCM/index.php/Home/Index/cf_details.html?id=3109), [3-Carboxy-4-Hydroxy-Phenoxy Glucoside](http://www.tcmip.cn/ETCM/index.php/Home/Index/cf_details.html?id=3121), [Columbamine](http://www.tcmip.cn/ETCM/index.php/Home/Index/cf_details.html?id=3122), [3,4-Dihydroxyphenylethyl Alcohol Glucoside](http://www.tcmip.cn/ETCM/index.php/Home/Index/cf_details.html?id=3123), [Epiberberine](http://www.tcmip.cn/ETCM/index.php/Home/Index/cf_details.html?id=3124), [2,3,4-Trihydroxy-Benzenepropanoic Acid](http://www.tcmip.cn/ETCM/index.php/Home/Index/cf_details.html?id=3125), [Groenlandicin](http://www.tcmip.cn/ETCM/index.php/Home/Index/cf_details.html?id=3126) (sedative and hypnotic, dilating coronary vessels, antipyretic and cooling, lowering blood pressure, anti-arrhythmic, stimulating smooth muscle of the gastrointestinal tract, enhancing hypoxia tolerance, anti-platelet aggregation, anti-gastric damage and anti-ulcer, promoting bile secretion, anti-radiation, lowering blood sugar, anti-inflammatory, anti-bacterial, anti-viral, anti-fungal, anti-tumor)  8.*Cornus officinalis Siebold* & Zucc. [Cornaceae; Corni Fructus]:  Cornus officinalis glycoside (also known as verbenin), senoside, saponin, tannin, vitamin A-like substances, gallic acid, fruit acid, tartaric acid (strengthening the heart, anti-platelet aggregation, dilating peripheral blood vessels, enhancing hypoxia tolerance, anti-shock, liver protection, anti-stress, diuresis, lowering blood sugar, immune regulation, anti-inflammatory, anti-bacterial, anti-fungal, anti-tumor)   1. *Eclipta prostrata (L.) L.* [Asteraceae; Ecliptae Herba]:   [2-(Buta-1,3-diynyl)-5-(4-chloro-3-hydroxybut-1-ynyl) thiophene](http://www.tcmip.cn/ETCM/index.php/Home/Index/cf_details.html?id=7314), [alpha-Terithenyl acetate](http://www.tcmip.cn/ETCM/index.php/Home/Index/cf_details.html?id=7315), [alpha-Terthienyl methanol](http://www.tcmip.cn/ETCM/index.php/Home/Index/cf_details.html?id=7316), [Butein](http://www.tcmip.cn/ETCM/index.php/Home/Index/cf_details.html?id=7317), [Butin](http://www.tcmip.cn/ETCM/index.php/Home/Index/cf_details.html?id=7318), [Chloromaloside](http://www.tcmip.cn/ETCM/index.php/Home/Index/cf_details.html?id=7319), [Demethylwedelolactone-7-glucoside](http://www.tcmip.cn/ETCM/index.php/Home/Index/cf_details.html?id=7320), [Demissine](http://www.tcmip.cn/ETCM/index.php/Home/Index/cf_details.html?id=7321), [Ecliptasaponin B](http://www.tcmip.cn/ETCM/index.php/Home/Index/cf_details.html?id=7322), [Edulinine](http://www.tcmip.cn/ETCM/index.php/Home/Index/cf_details.html?id=7323), [Isodesacetyluvaricin](http://www.tcmip.cn/ETCM/index.php/Home/Index/cf_details.html?id=7324), [Niacin,Nicotinic Acid](http://www.tcmip.cn/ETCM/index.php/Home/Index/cf_details.html?id=1382), [Testosterone](http://www.tcmip.cn/ETCM/index.php/Home/Index/cf_details.html?id=7325), [PHB](http://www.tcmip.cn/ETCM/index.php/Home/Index/cf_details.html?id=7297),[3,4-Dihydroxybenzoic Acid,Protocatechuic Acid](http://www.tcmip.cn/ETCM/index.php/Home/Index/cf_details.html?id=406), [Acacini,Linarin](http://www.tcmip.cn/ETCM/index.php/Home/Index/cf_details.html?id=4203), [Acacetin](http://www.tcmip.cn/ETCM/index.php/Home/Index/cf_details.html?id=2245), [Hexahydrofarnesyl Acetone](http://www.tcmip.cn/ETCM/index.php/Home/Index/cf_details.html?id=164), [Cinaroside](http://www.tcmip.cn/ETCM/index.php/Home/Index/cf_details.html?id=3023), [(1R,2R,4R)-Dihydrocarveol](http://www.tcmip.cn/ETCM/index.php/Home/Index/cf_details.html?id=7326), [.alpha.-T COOH deriv.](http://www.tcmip.cn/ETCM/index.php/Home/Index/cf_details.html?id=7327), [1,3,8,9-tetrahydroxybenzofurano[3,2-c]chromen-6-one](http://www.tcmip.cn/ETCM/index.php/Home/Index/cf_details.html?id=7328), [Ecliptasaponin D_qt](http://www.tcmip.cn/ETCM/index.php/Home/Index/cf_details.html?id=7329), [Ecliptasaponin](http://www.tcmip.cn/ETCM/index.php/Home/Index/cf_details.html?id=7330), [3-[(2S)-2,3-dihydroxy-3-methyl-butyl]-4-methoxy-1-methyl-carbostyril](http://www.tcmip.cn/ETCM/index.php/Home/Index/cf_details.html?id=7331), [3'-O-Methylorobol](http://www.tcmip.cn/ETCM/index.php/Home/Index/cf_details.html?id=7332), [TES](http://www.tcmip.cn/ETCM/index.php/Home/Index/cf_details.html?id=7333), [Î’-Guaiene](http://www.tcmip.cn/ETCM/index.php/Home/Index/cf_details.html?id=6698), [Benzofurano(3',2':3,4)coumarin](http://www.tcmip.cn/ETCM/index.php/Home/Index/cf_details.html?id=7334), [(3S,8S,9S,10R,13R,14S,17R)-17-[(1R,4R)-1,4-dimethylhexyl]-10,13-dimethyl-2,3,4,7,8,9,11,12,14,15,16,17-dodecahydro-1H-cyclopenta[a]phenanthren-3-ol](http://www.tcmip.cn/ETCM/index.php/Home/Index/cf_details.html?id=7335), [echinocystic acid](http://www.tcmip.cn/ETCM/index.php/Home/Index/cf_details.html?id=7336), [(3S,8S,9S,10R,13R,14S,17R)-17-[(E,1R,4R)-1,4-dimethylhex-2-enyl]-10,13-dimethyl-2,3,4,7,8,9,11,12,14,15,16,17-dodecahydro-1H-cyclopenta[a]phenanthren-3-ol](http://www.tcmip.cn/ETCM/index.php/Home/Index/cf_details.html?id=7337), [Pratensein](http://www.tcmip.cn/ETCM/index.php/Home/Index/cf_details.html?id=5707), [Caulophyllogenin](http://www.tcmip.cn/ETCM/index.php/Home/Index/cf_details.html?id=7338)  [(X{2212})-Nicotine](http://www.tcmip.cn/ETCM/index.php/Home/Index/cf_details.html?id=1959), [wedelolactone](http://www.tcmip.cn/ETCM/index.php/Home/Index/cf_details.html?id=7339), [Î’-Amyrin](http://www.tcmip.cn/ETCM/index.php/Home/Index/cf_details.html?id=71), [(-)-Caryophyllene Oxide,(1R,4R,6R,10S)-4,12,12-Trimethyl-9-Methylidene-5-Oxatricyclo[8.2.0.0~4,6~]Dodecane](http://www.tcmip.cn/ETCM/index.php/Home/Index/cf_details.html?id=6529), [Luteolin](http://www.tcmip.cn/ETCM/index.php/Home/Index/cf_details.html?id=841), [Apigenin-7-O-Glucoside,Apigetrin,Cosmosiin](http://www.tcmip.cn/ETCM/index.php/Home/Index/cf_details.html?id=5548), [Apigenin](http://www.tcmip.cn/ETCM/index.php/Home/Index/cf_details.html?id=372), [Guercetol,Quercetin,Quercetin,Sophoretin,Meletin,Xanthaurine](http://www.tcmip.cn/ETCM/index.php/Home/Index/cf_details.html?id=93) (Sedation and hypnosis, analgesia, hemostasis, promoting blood coagulation, anti-myocardial ischemia, liver protection, immune regulation, anti-bacterial, anti-tumor, anti-mutation)   1. *Rhus chinensis* Mill. [Anacardiaceae; Galla Chinensis]:   Gallnut tannic acid, gallic acid, fat, resin, starch and wax (anti-fertility, anti-bacterial, anti-viral, anti-tumor, astringent) |
| Shen Zhen | Tianqi Jiangtang Capsules (Specification: 0.32 g/capsule) | Heilongjiang Weiming Tianren Pharmaceutical Co., Ltd | - *Astragalus membranaceus* Fisch. ex Bunge [Fabaceae; Astragali Radix], root and rhizome. - *Trichosanthes kirilowii* Maxim. [Cucurbitaceae; Trichosanthis Radix], root - *Ligustrum lucidum* W.T.Aiton [Oleaceae; Ligustri Lucidi Fructus], ripe fruit. - *Dendrobium nobile* Lindl. [Orchidaceae; Dendrobii Caulis], stem. - *Panax ginseng* C.A.Mey. [Araliaceae; Ginseng Radix et Rhizoma], root. - *Lycium chinense* Mill. [Solanaceae; Lycii Cortex], dried root bark. - *Coptis chinensis* Franch. [Ranumculaceae; Coptidis Rhizoma], rhizome. - *Cornus officinalis* Siebold & Zucc. [Cornaceae; Corni Fructus], ripe pulp. - *Eclipta prostrata (L.) L.* [Asteraceae; Ecliptae Herba], dried Above-Ground Part. - *Rhus chinensis* Mill. [Anacardiaceae; Galla Chinensis], Galls on the leaves. | Y – Prepared according to National Drug Standards of China Food and Drug Administration (Z20063799) | N | N |  |
| Yang Qingping | Tianqi Jiangtang Capsules (Specification: 0.32 g/capsule) | Heilongjiang Weiming Tianren Pharmaceutical Co., Ltd | - *Astragalus membranaceus* Fisch. ex Bunge [Fabaceae; Astragali Radix], root and rhizome. - *Trichosanthes kirilowii* Maxim. [Cucurbitaceae; Trichosanthis Radix], root - *Ligustrum lucidum* W.T.Aiton [Oleaceae; Ligustri Lucidi Fructus], ripe fruit. - *Dendrobium nobile* Lindl. [Orchidaceae; Dendrobii Caulis], stem. - *Panax ginseng* C.A.Mey. [Araliaceae; Ginseng Radix et Rhizoma], root. - *Lycium chinense* Mill. [Solanaceae; Lycii Cortex], dried root bark. - *Coptis chinensis* Franch. [Ranumculaceae; Coptidis Rhizoma], rhizome. - *Cornus officinalis* Siebold & Zucc. [Cornaceae; Corni Fructus], ripe pulp. - *Eclipta prostrata (L.) L.* [Asteraceae; Ecliptae Herba], dried Above-Ground Part. - *Rhus chinensis* Mill. [Anacardiaceae; Galla Chinensis], Galls on the leaves. | N | Y-130903, 151207 | N |  |
| Qao Hongwei | Tianqi Jiangtang Capsules (Specification: 0.32 g/capsule) | Heilongjiang Baoquan Pharmaceutical Co., Ltd | - *Astragalus membranaceus* Fisch. ex Bunge [Fabaceae; Astragali Radix], root and rhizome. - *Trichosanthes kirilowii* Maxim. [Cucurbitaceae; Trichosanthis Radix], root - *Ligustrum lucidum* W.T.Aiton [Oleaceae; Ligustri Lucidi Fructus], ripe fruit. - *Dendrobium nobile* Lindl. [Orchidaceae; Dendrobii Caulis], stem. - *Panax ginseng* C.A.Mey. [Araliaceae; Ginseng Radix et Rhizoma], root. - *Lycium chinense* Mill. [Solanaceae; Lycii Cortex], dried root bark. - *Coptis chinensis* Franch. [Ranumculaceae; Coptidis Rhizoma], rhizome. - *Cornus officinalis* Siebold & Zucc. [Cornaceae; Corni Fructus], ripe pulp. - *Eclipta prostrata (L.) L.* [Asteraceae; Ecliptae Herba], dried Above-Ground Part. - *Rhus chinensis* Mill. [Anacardiaceae; Galla Chinensis], Galls on the leaves. | Y – Prepared according to National Drug Standards of China Food and Drug Administration (Z20063799) | N | N |  |
| Xu Sui | Tianqi Jiangtang Capsules (Specification: 0.32 g/capsule) | Heilongjiang Weiming Tianren Pharmaceutical Co., Ltd | - *Astragalus membranaceus* Fisch. ex Bunge [Fabaceae; Astragali Radix], root and rhizome. - *Trichosanthes kirilowii* Maxim. [Cucurbitaceae; Trichosanthis Radix], root - *Ligustrum lucidum* W.T.Aiton [Oleaceae; Ligustri Lucidi Fructus], ripe fruit. - *Dendrobium nobile* Lindl. [Orchidaceae; Dendrobii Caulis], stem. - *Panax ginseng* C.A.Mey. [Araliaceae; Ginseng Radix et Rhizoma], root. - *Lycium chinense* Mill. [Solanaceae; Lycii Cortex], dried root bark. - *Coptis chinensis* Franch. [Ranumculaceae; Coptidis Rhizoma], rhizome. - *Cornus officinalis* Siebold & Zucc. [Cornaceae; Corni Fructus], ripe pulp. - *Eclipta prostrata (L.) L.* [Asteraceae; Ecliptae Herba], dried Above-Ground Part. - *Rhus chinensis* Mill. [Anacardiaceae; Galla Chinensis], Galls on the leaves. | N | Y-160307 | N |  |
| Chai Hong | Tianqi Jiangtang Capsules (Specification: 0.32 g/capsule) |  | - *Astragalus membranaceus* Fisch. ex Bunge [Fabaceae; Astragali Radix], root and rhizome. - *Trichosanthes kirilowii* Maxim. [Cucurbitaceae; Trichosanthis Radix], root - *Ligustrum lucidum* W.T.Aiton [Oleaceae; Ligustri Lucidi Fructus], ripe fruit. - *Dendrobium nobile* Lindl. [Orchidaceae; Dendrobii Caulis], stem. - *Panax ginseng* C.A.Mey. [Araliaceae; Ginseng Radix et Rhizoma], root. - *Lycium chinense* Mill. [Solanaceae; Lycii Cortex], dried root bark. - *Coptis chinensis* Franch. [Ranumculaceae; Coptidis Rhizoma], rhizome. - *Cornus officinalis* Siebold & Zucc. [Cornaceae; Corni Fructus], ripe pulp. - *Eclipta prostrata (L.) L.* [Asteraceae; Ecliptae Herba], dried Above-Ground Part. - *Rhus chinensis* Mill. [Anacardiaceae; Galla Chinensis], Galls on the leaves. | Y – Prepared according to National Drug Standards of China Food and Drug Administration (Z20063799) | N | N |  |
| Hou Cuirong | Tianqi Jiangtang Capsules (Specification: 0.32 g/capsule) | Heilongjiang Weiming Tianren Pharmaceutical Co., Ltd | - *Astragalus membranaceus* Fisch. ex Bunge [Fabaceae; Astragali Radix], root and rhizome. - *Trichosanthes kirilowii* Maxim. [Cucurbitaceae; Trichosanthis Radix], root - *Ligustrum lucidum* W.T.Aiton [Oleaceae; Ligustri Lucidi Fructus], ripe fruit. - *Dendrobium nobile* Lindl. [Orchidaceae; Dendrobii Caulis], stem. - *Panax ginseng* C.A.Mey. [Araliaceae; Ginseng Radix et Rhizoma], root. - *Lycium chinense* Mill. [Solanaceae; Lycii Cortex], dried root bark. - *Coptis chinensis* Franch. [Ranumculaceae; Coptidis Rhizoma], rhizome. - *Cornus officinalis* Siebold & Zucc. [Cornaceae; Corni Fructus], ripe pulp. - *Eclipta prostrata (L.) L.* [Asteraceae; Ecliptae Herba], dried Above-Ground Part. - *Rhus chinensis* Mill. [Anacardiaceae; Galla Chinensis], Galls on the leaves. | Y – Prepared according to National Drug Standards of China Food and Drug Administration (Z20063799) | N | N |  |
| Hou Guangming | Tianqi Jiangtang Capsules (Specification: 0.32 g/capsule) | Heilongjiang Weiming Tianren Pharmaceutical Co., Ltd | - *Astragalus membranaceus* Fisch. ex Bunge [Fabaceae; Astragali Radix], root and rhizome. - *Trichosanthes kirilowii* Maxim. [Cucurbitaceae; Trichosanthis Radix], root - *Ligustrum lucidum* W.T.Aiton [Oleaceae; Ligustri Lucidi Fructus], ripe fruit. - *Dendrobium nobile* Lindl. [Orchidaceae; Dendrobii Caulis], stem. - *Panax ginseng* C.A.Mey. [Araliaceae; Ginseng Radix et Rhizoma], root. - *Lycium chinense* Mill. [Solanaceae; Lycii Cortex], dried root bark. - *Coptis chinensis* Franch. [Ranumculaceae; Coptidis Rhizoma], rhizome. - *Cornus officinalis* Siebold & Zucc. [Cornaceae; Corni Fructus], ripe pulp. - *Eclipta prostrata (L.) L.* [Asteraceae; Ecliptae Herba], dried Above-Ground Part. - *Rhus chinensis* Mill. [Anacardiaceae; Galla Chinensis], Galls on the leaves. | Y – Prepared according to National Drug Standards of China Food and Drug Administration (Z20063799) | N | N |  |
| Wu Feng | Tianqi Jiangtang Capsules (Specification: 0.32 g/capsule) | Heilongjiang Weiming Tianren Pharmaceutical Co., Ltd | - *Astragalus membranaceus* Fisch. ex Bunge [Fabaceae; Astragali Radix], root and rhizome. - *Trichosanthes kirilowii* Maxim. [Cucurbitaceae; Trichosanthis Radix], root - *Ligustrum lucidum* W.T.Aiton [Oleaceae; Ligustri Lucidi Fructus], ripe fruit. - *Dendrobium nobile* Lindl. [Orchidaceae; Dendrobii Caulis], stem. - *Panax ginseng* C.A.Mey. [Araliaceae; Ginseng Radix et Rhizoma], root. - *Lycium chinense* Mill. [Solanaceae; Lycii Cortex], dried root bark. - *Coptis chinensis* Franch. [Ranumculaceae; Coptidis Rhizoma], rhizome. - *Cornus officinalis* Siebold & Zucc. [Cornaceae; Corni Fructus], ripe pulp. - *Eclipta prostrata (L.) L.* [Asteraceae; Ecliptae Herba], dried Above-Ground Part. - *Rhus chinensis* Mill. [Anacardiaceae; Galla Chinensis], Galls on the leaves. | N | Y-151225 | N |  |
| Wu Huachen | Tianqi Jiangtang Capsules (Specification: 0.32 g/capsule) | Heilongjiang Weiming Tianren Pharmaceutical Co., Ltd | - *Astragalus membranaceus* Fisch. ex Bunge [Fabaceae; Astragali Radix], root and rhizome. - *Trichosanthes kirilowii* Maxim. [Cucurbitaceae; Trichosanthis Radix], root - *Ligustrum lucidum* W.T.Aiton [Oleaceae; Ligustri Lucidi Fructus], ripe fruit. - *Dendrobium nobile* Lindl. [Orchidaceae; Dendrobii Caulis], stem. - *Panax ginseng* C.A.Mey. [Araliaceae; Ginseng Radix et Rhizoma], root. - *Lycium chinense* Mill. [Solanaceae; Lycii Cortex], dried root bark. - *Coptis chinensis* Franch. [Ranumculaceae; Coptidis Rhizoma], rhizome. - *Cornus officinalis* Siebold & Zucc. [Cornaceae; Corni Fructus], ripe pulp. - *Eclipta prostrata (L.) L.* [Asteraceae; Ecliptae Herba], dried Above-Ground Part. - *Rhus chinensis* Mill. [Anacardiaceae; Galla Chinensis], Galls on the leaves. | Y – Prepared according to National Drug Standards of China Food and Drug Administration (Z20063799) | N | N |  |
| Tang Xianyu | Tianqi Jiangtang Capsules (Specification: 0.32 g/capsule) | Heilongjiang Weiming Tianren Pharmaceutical Co., Ltd | - *Astragalus membranaceus* Fisch. ex Bunge [Fabaceae; Astragali Radix], root and rhizome. - *Trichosanthes kirilowii* Maxim. [Cucurbitaceae; Trichosanthis Radix], root - *Ligustrum lucidum* W.T.Aiton [Oleaceae; Ligustri Lucidi Fructus], ripe fruit. - *Dendrobium nobile* Lindl. [Orchidaceae; Dendrobii Caulis], stem. - *Panax ginseng* C.A.Mey. [Araliaceae; Ginseng Radix et Rhizoma], root. - *Lycium chinense* Mill. [Solanaceae; Lycii Cortex], dried root bark. - *Coptis chinensis* Franch. [Ranumculaceae; Coptidis Rhizoma], rhizome. - *Cornus officinalis* Siebold & Zucc. [Cornaceae; Corni Fructus], ripe pulp. - *Eclipta prostrata (L.) L.* [Asteraceae; Ecliptae Herba], dried Above-Ground Part. - *Rhus chinensis* Mill. [Anacardiaceae; Galla Chinensis], Galls on the leaves. | N | N | N |  |
| Cao Yong | Tianqi Jiangtang Capsules (Specification: 0.32 g/capsule) | Heilongjiang Baoquan Pharmaceutical Co., Ltd | - *Astragalus membranaceus* Fisch. ex Bunge [Fabaceae; Astragali Radix], root and rhizome. - *Trichosanthes kirilowii* Maxim. [Cucurbitaceae; Trichosanthis Radix], root - *Ligustrum lucidum* W.T.Aiton [Oleaceae; Ligustri Lucidi Fructus], ripe fruit. - *Dendrobium nobile* Lindl. [Orchidaceae; Dendrobii Caulis], stem. - *Panax ginseng* C.A.Mey. [Araliaceae; Ginseng Radix et Rhizoma], root. - *Lycium chinense* Mill. [Solanaceae; Lycii Cortex], dried root bark. - *Coptis chinensis* Franch. [Ranumculaceae; Coptidis Rhizoma], rhizome. - *Cornus officinalis* Siebold & Zucc. [Cornaceae; Corni Fructus], ripe pulp. - *Eclipta prostrata (L.) L.* [Asteraceae; Ecliptae Herba], dried Above-Ground Part. - *Rhus chinensis* Mill. [Anacardiaceae; Galla Chinensis], Galls on the leaves. | Y – Prepared according to National Drug Standards of China Food and Drug Administration (Z20063799) | N | N |  |
| Lian Fengmei | Tianqi Jiangtang Capsules (Specification: 0.32 g/capsule) | Heilongjiang Baoquan Pharmaceutical Co., Ltd | - *Astragalus membranaceus* Fisch. ex Bunge [Fabaceae; Astragali Radix], root and rhizome. - *Trichosanthes kirilowii* Maxim. [Cucurbitaceae; Trichosanthis Radix], root - *Ligustrum lucidum* W.T.Aiton [Oleaceae; Ligustri Lucidi Fructus], ripe fruit. - *Dendrobium nobile* Lindl. [Orchidaceae; Dendrobii Caulis], stem. - *Panax ginseng* C.A.Mey. [Araliaceae; Ginseng Radix et Rhizoma], root. - *Lycium chinense* Mill. [Solanaceae; Lycii Cortex], dried root bark. - *Coptis chinensis* Franch. [Ranumculaceae; Coptidis Rhizoma], rhizome. - *Cornus officinalis* Siebold & Zucc. [Cornaceae; Corni Fructus], ripe pulp. - *Eclipta prostrata (L.) L.* [Asteraceae; Ecliptae Herba], dried Above-Ground Part. - *Rhus chinensis* Mill. [Anacardiaceae; Galla Chinensis], Galls on the leaves. | N | N | N |  |

# Supplementary Figures


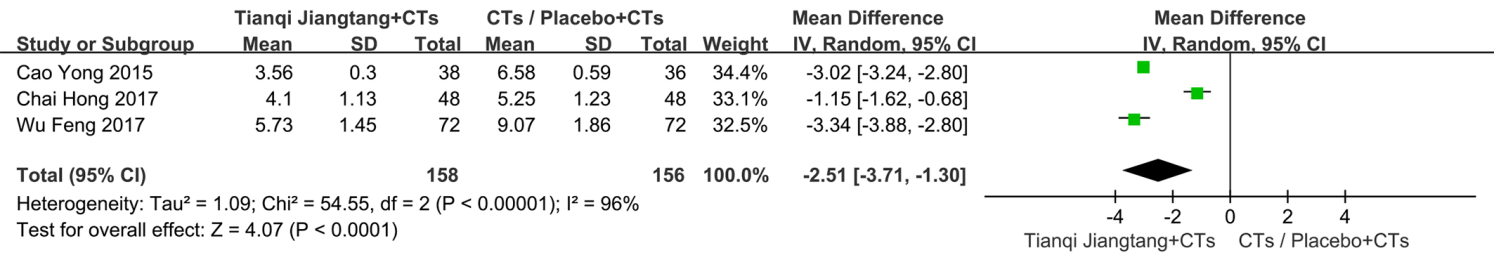


Fig. S1. Overall pooled results forest plot comparing Tianqi Jiangtang Capsule plus conventional treatments (CTs) to placebo plus CTs or CTs alone on hs-CRP.


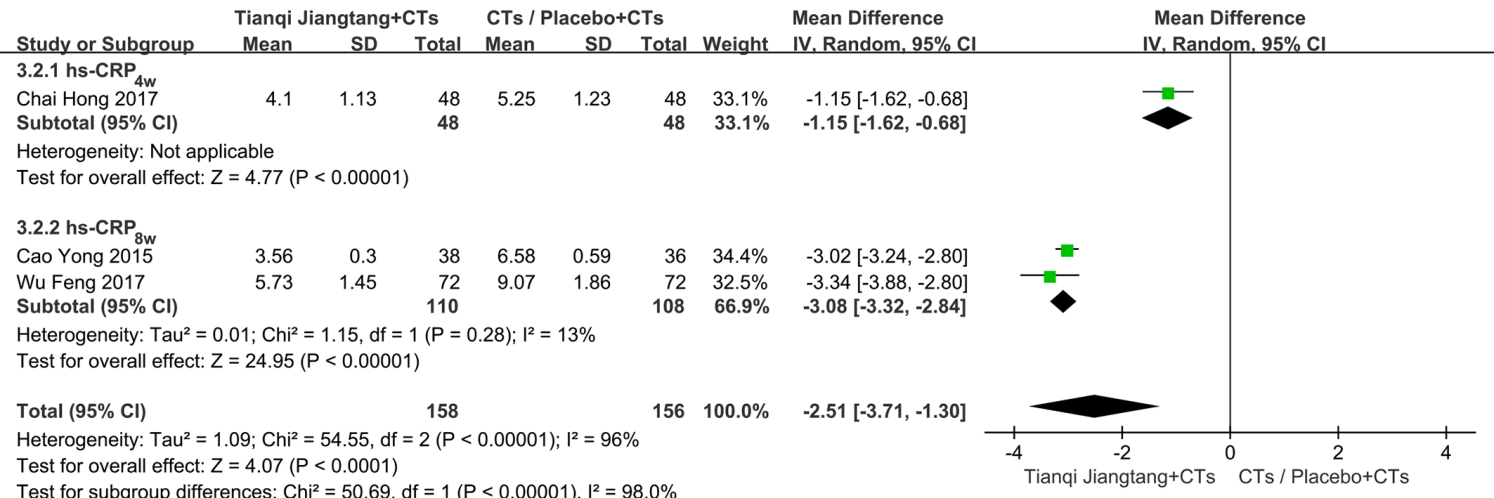


Fig. S2. Subgroup analysis forest plot comparing Tianqi Jiangtang Capsule plus conventional treatments (CTs) to placebo plus CTs or CTs alone on hs-CRP.


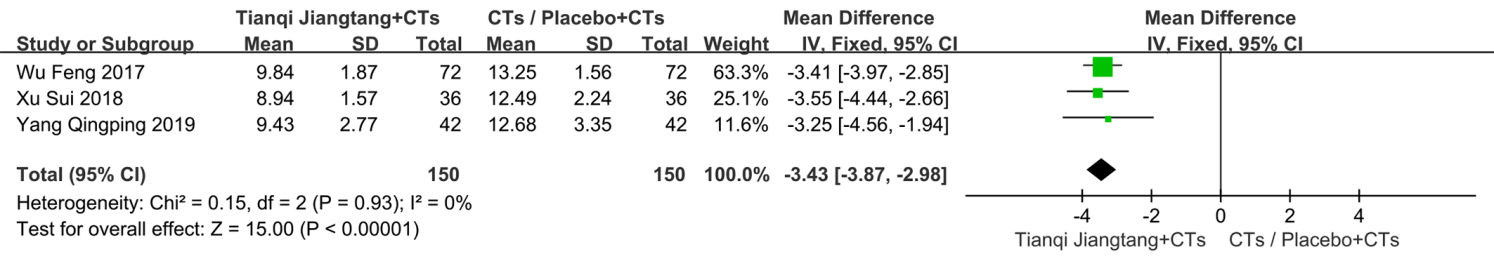


Fig. S3. Overall pooled results forest plot comparing Tianqi Jiangtang Capsule plus conventional treatments (CTs) to placebo plus CTs or CTs alone on IL-6.


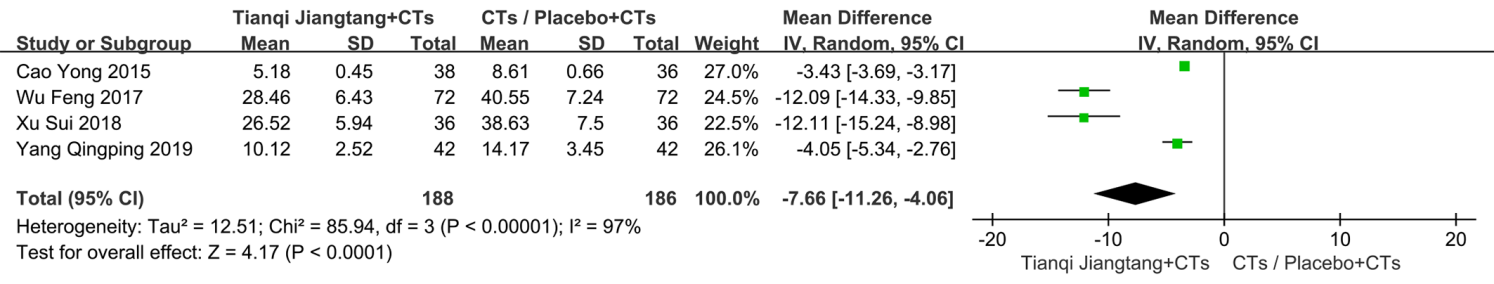


Fig. S4. Overall pooled results forest plot comparing Tianqi Jiangtang Capsule plus conventional treatments (CTs) to placebo plus CTs or CTs alone on TNF-α.


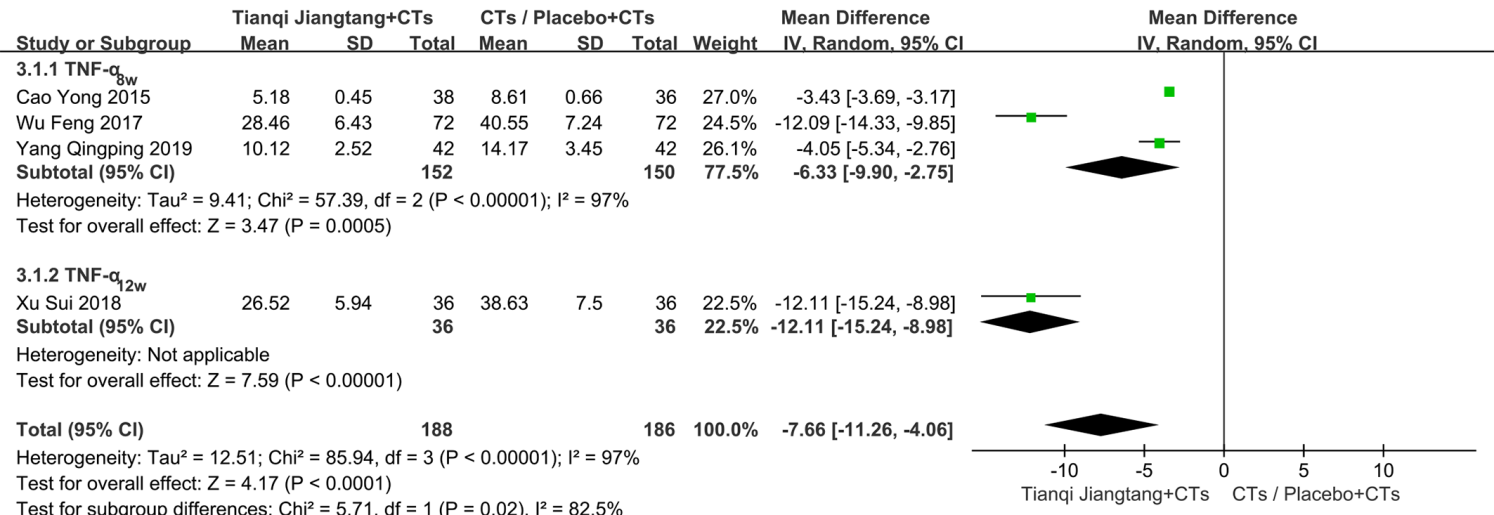


Fig. S5. Subgroup analysis forest plot comparing Tianqi Jiangtang Capsule plus conventional treatments (CTs) to placebo plus CTs or CTs alone on TNF-α.


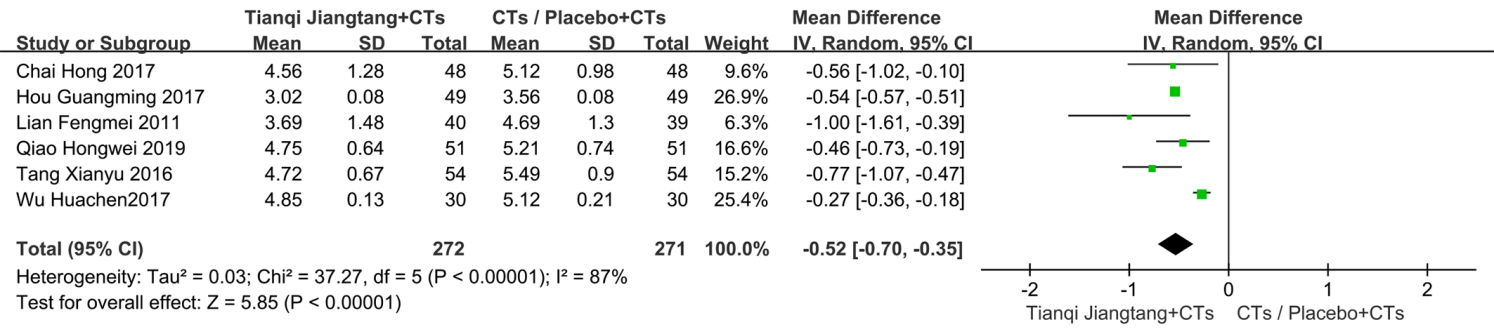


Fig. S6. Overall pooled results forest plot comparing Tianqi Jiangtang Capsule plus conventional treatments (CTs) to placebo plus CTs or CTs alone on TC.


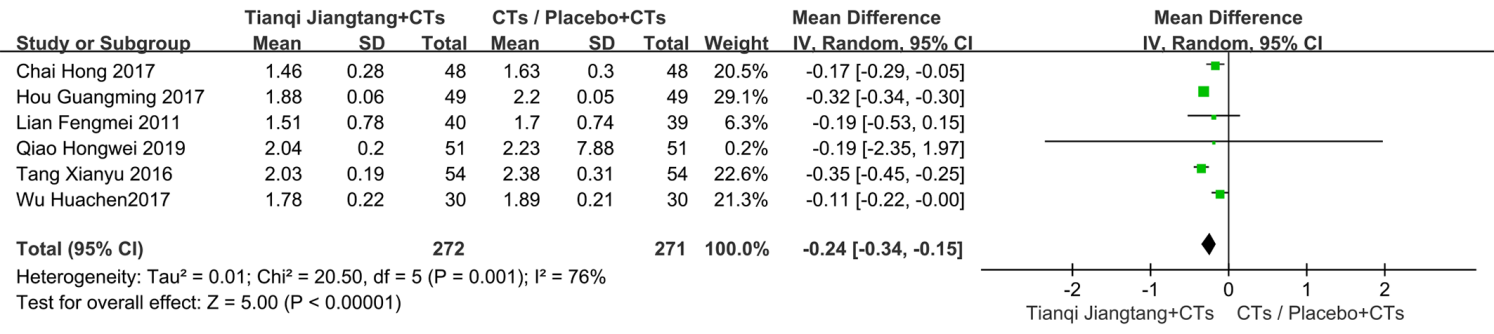


Fig. S7. Overall pooled results forest plot comparing Tianqi Jiangtang Capsule plus conventional treatments (CTs) to placebo plus CTs or CTs alone on TG.


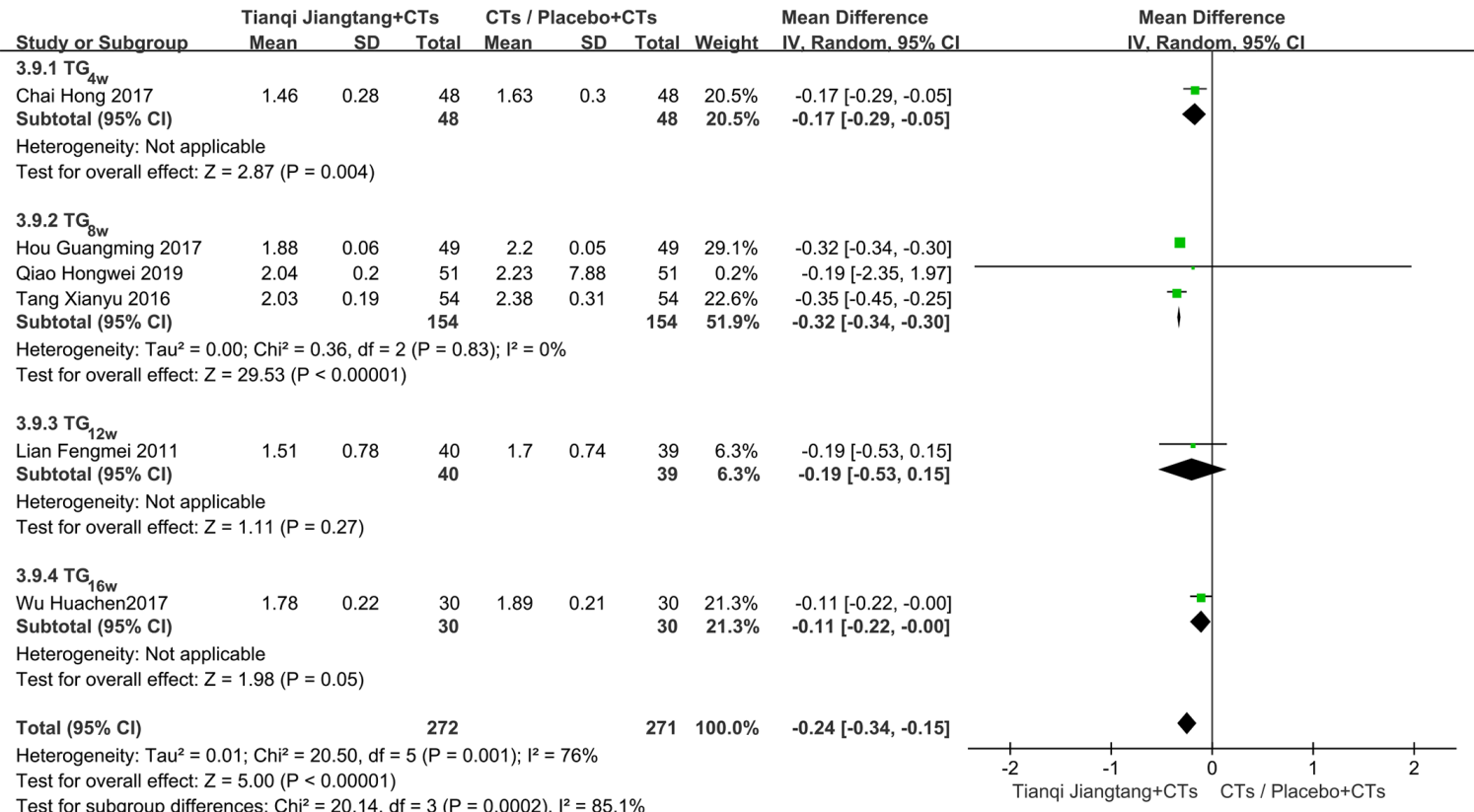


Fig. S8. Subgroup analysis forest plot comparing Tianqi Jiangtang Capsule plus conventional treatments (CTs) to placebo plus CTs or CTs alone on TG.


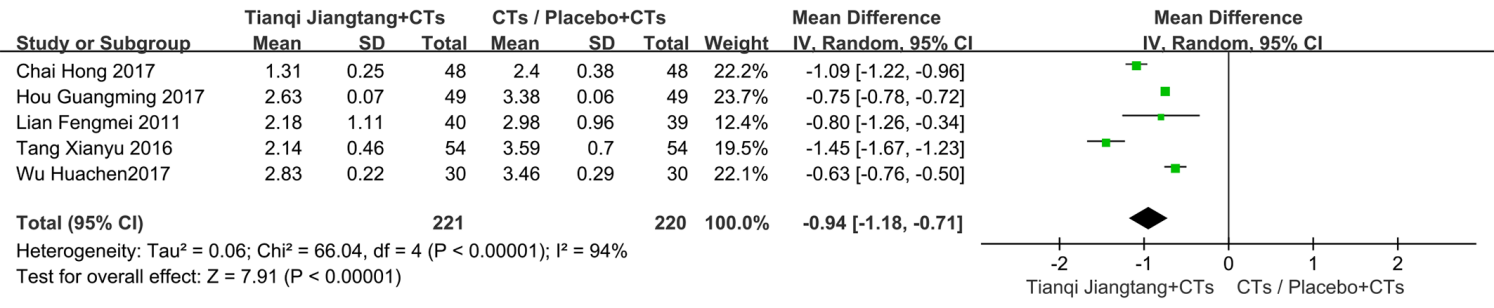


Fig. S9. Overall pooled results forest plot comparing Tianqi Jiangtang Capsule plus conventional treatments (CTs) to placebo plus CTs or CTs alone on LDL-C.


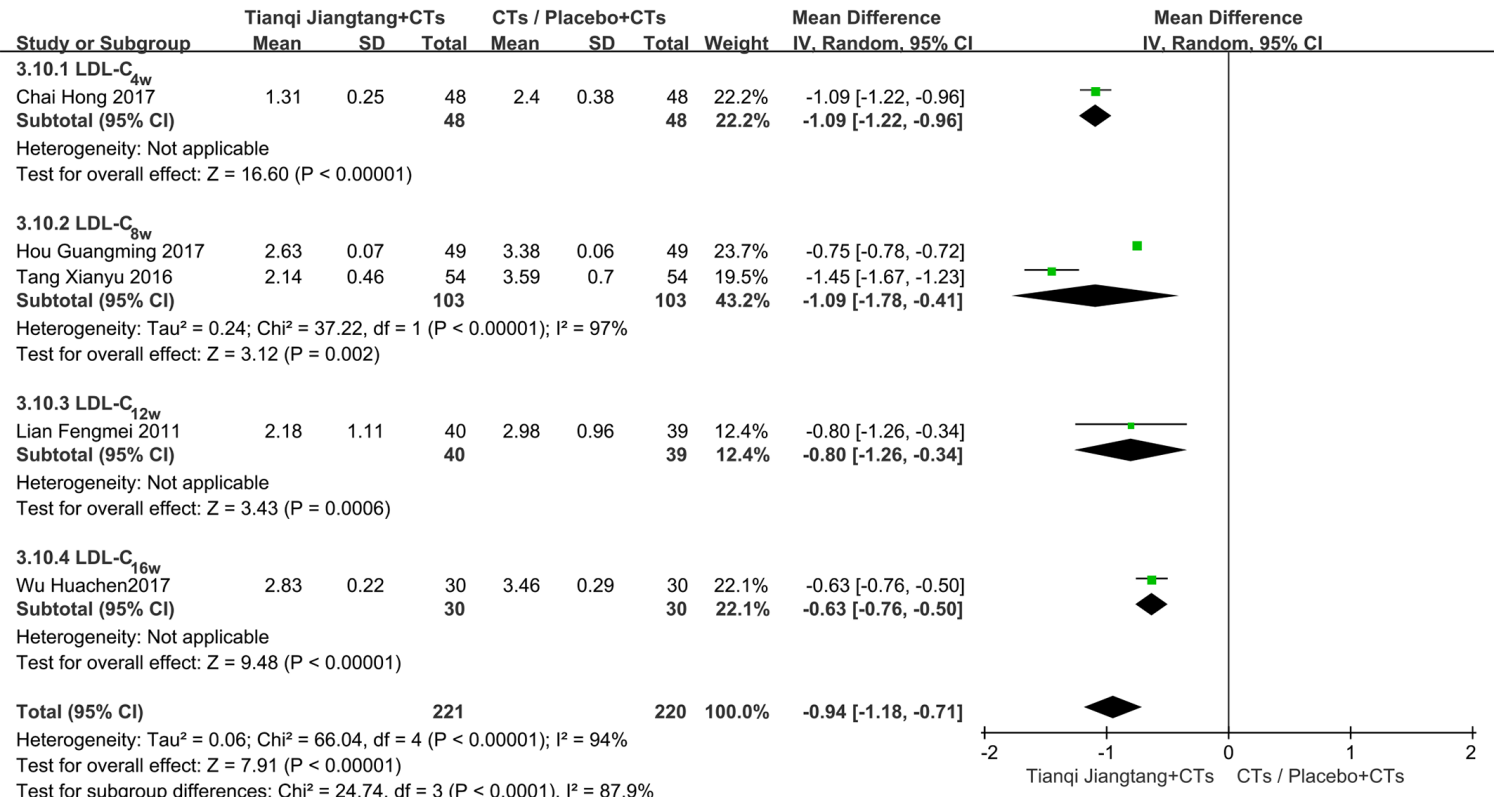


Fig. S10. Subgroup analysis forest plot comparing Tianqi Jiangtang Capsule plus conventional treatments (CTs) to placebo plus CTs or CTs alone on LDL-C.


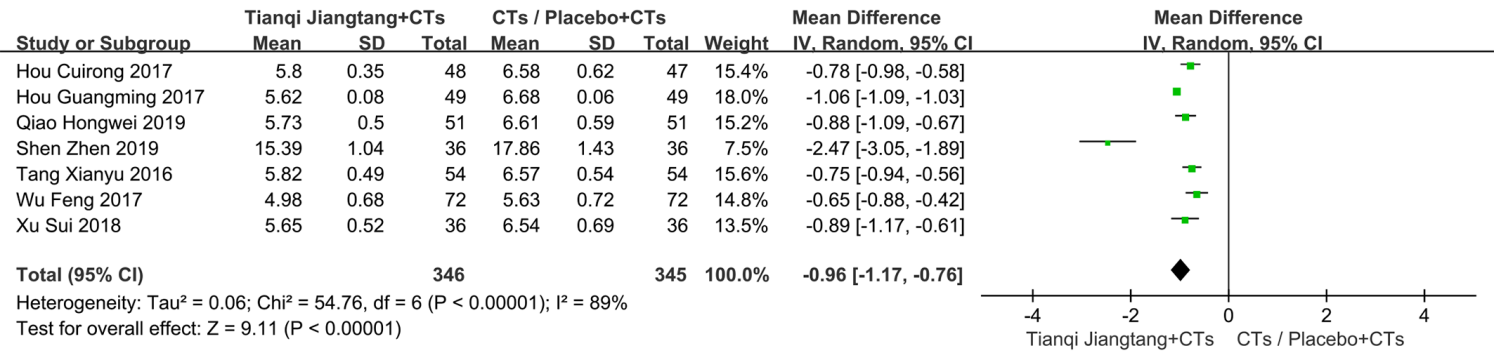


Fig. S11. Overall pooled results forest plot comparing Tianqi Jiangtang Capsule plus conventional treatments (CTs) to placebo plus CTs or CTs alone on BUN.


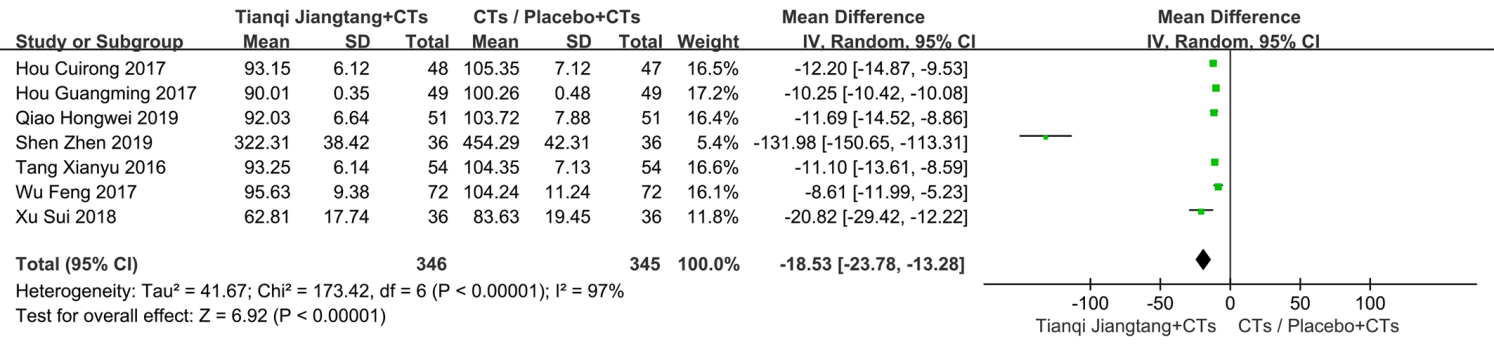


Fig. S12. Overall pooled results forest plot comparing Tianqi Jiangtang Capsule plus conventional treatments (CTs) to placebo plus CTs or CTs alone on Scr.


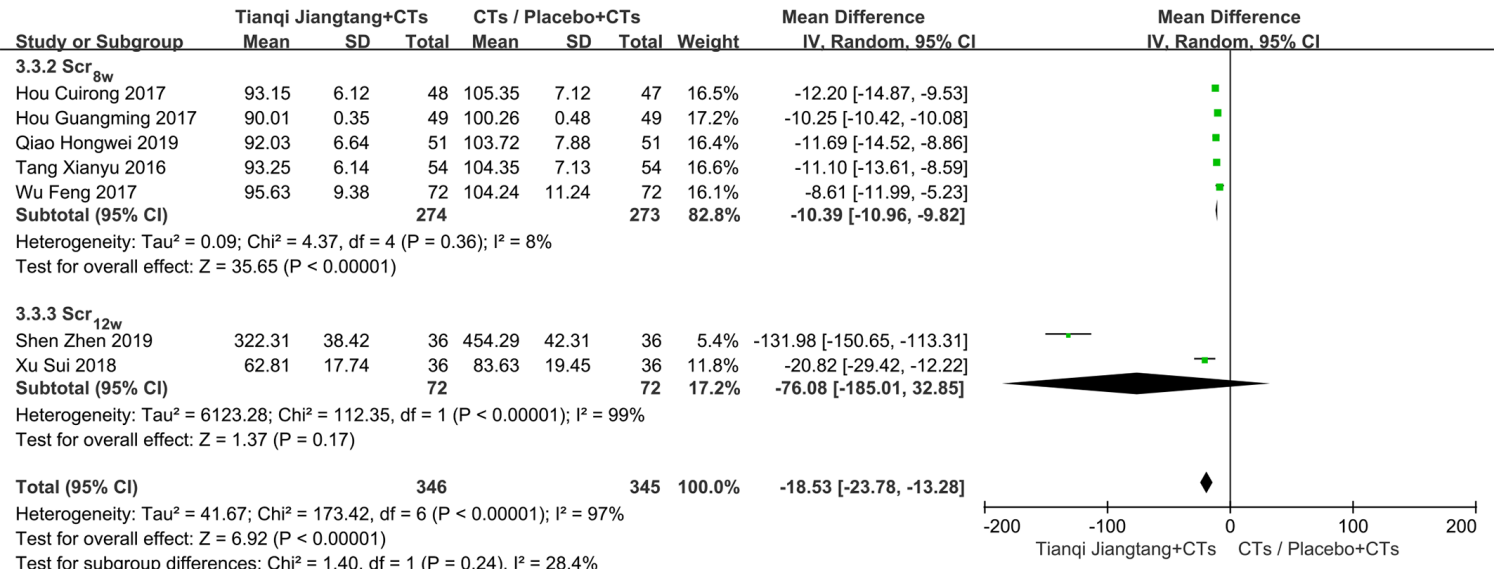


Fig. S13. Subgroup analysis forest plot comparing Tianqi Jiangtang Capsule plus conventional treatments (CTs) to placebo plus CTs or CTs alone on Scr.


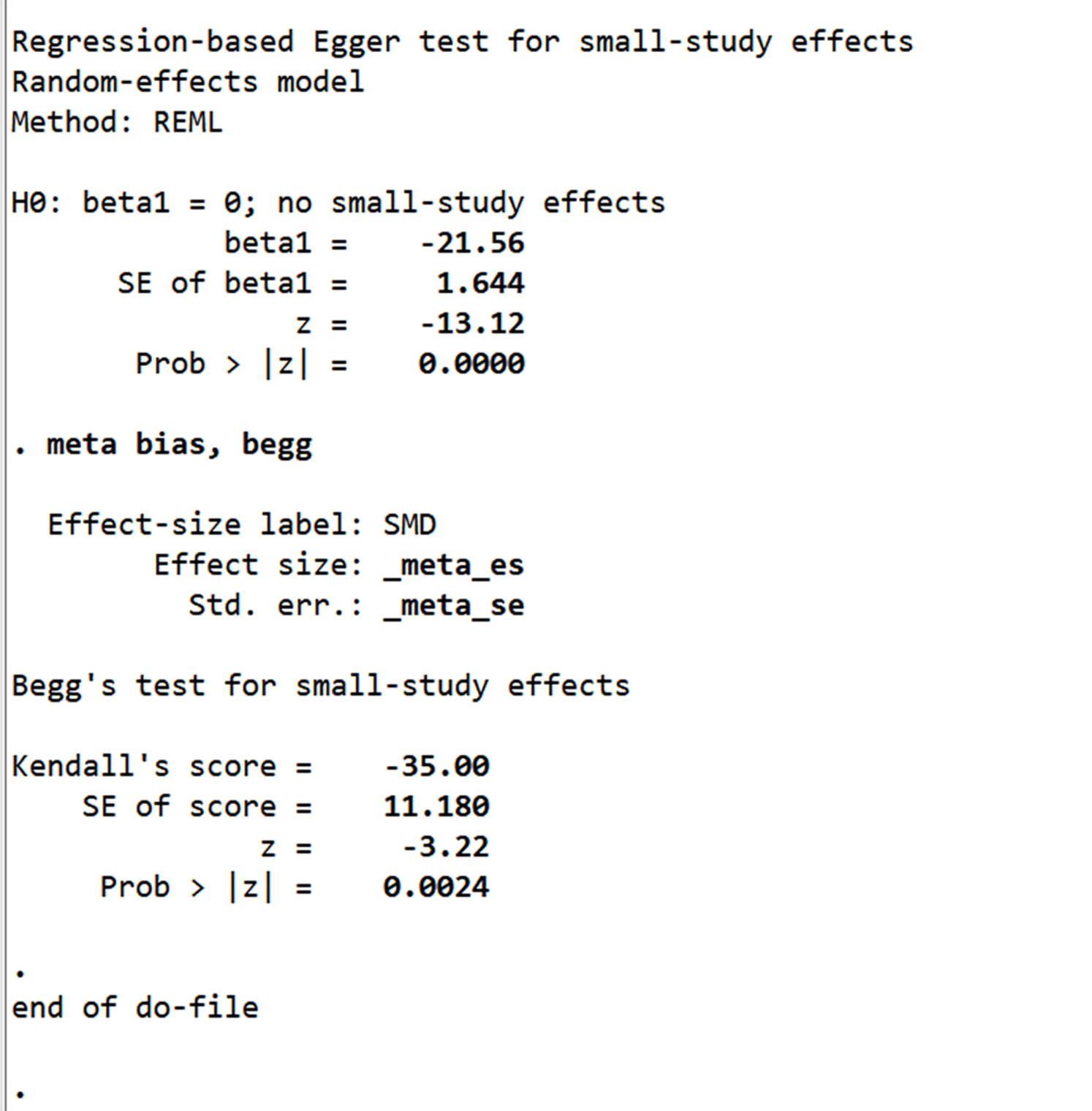


Fig. S14 Results of Egger's and Begg's tests on HbA1c.


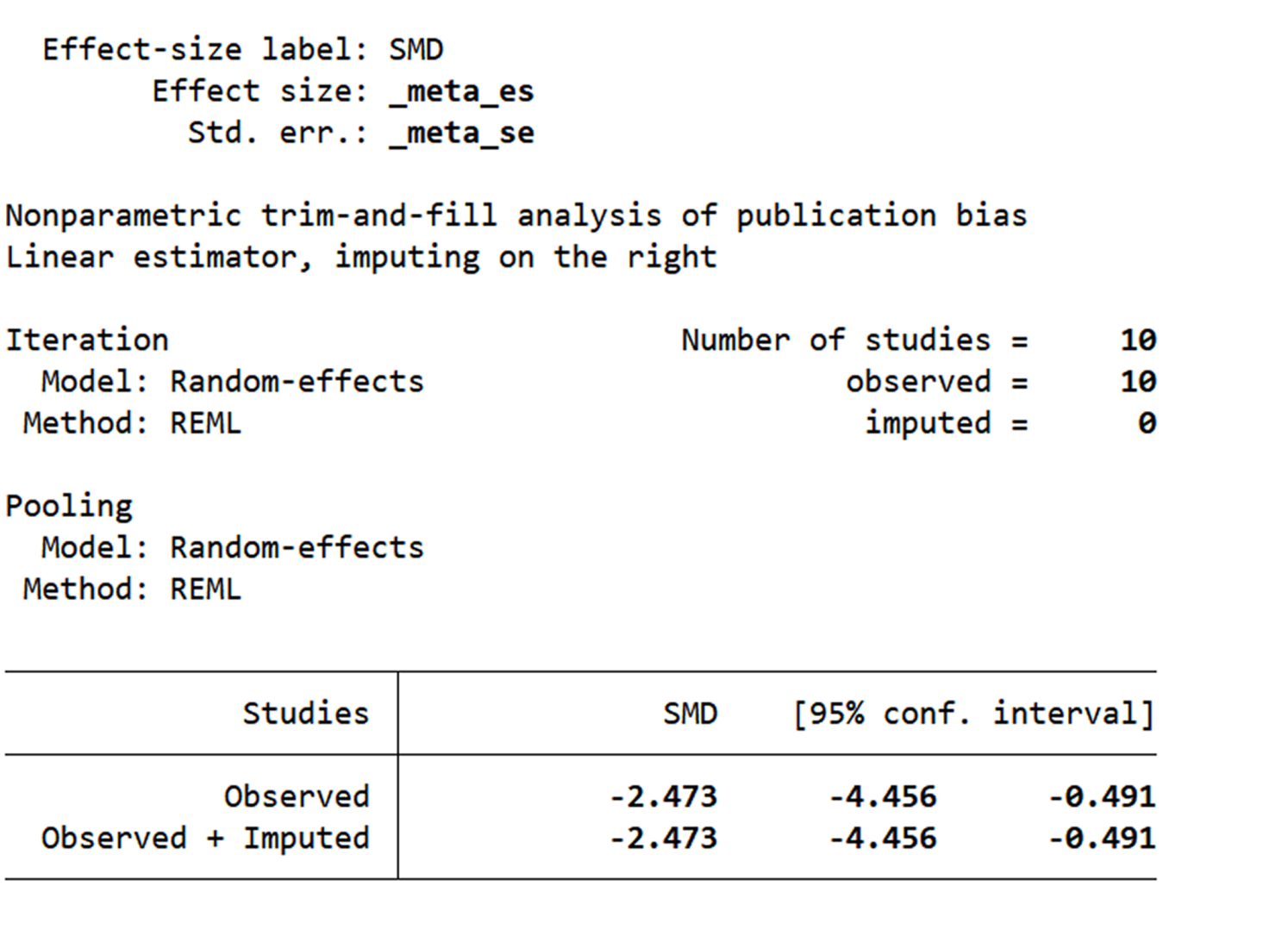


Fig. S15 Adjusted effect size of HbA1c.


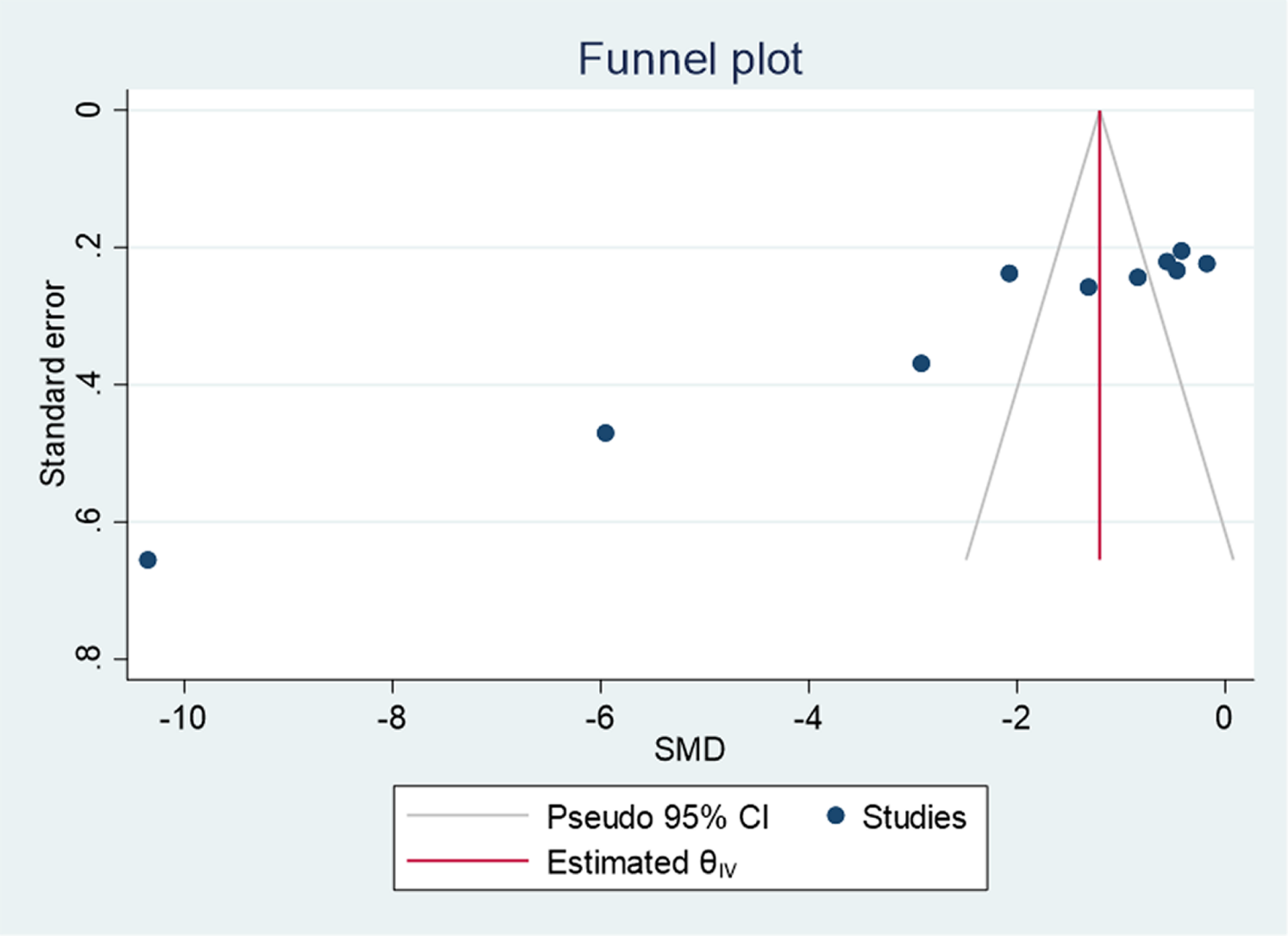


Fig. S16. Trim-and-fill adjusted funnel plot & egger plot of HbA1c.


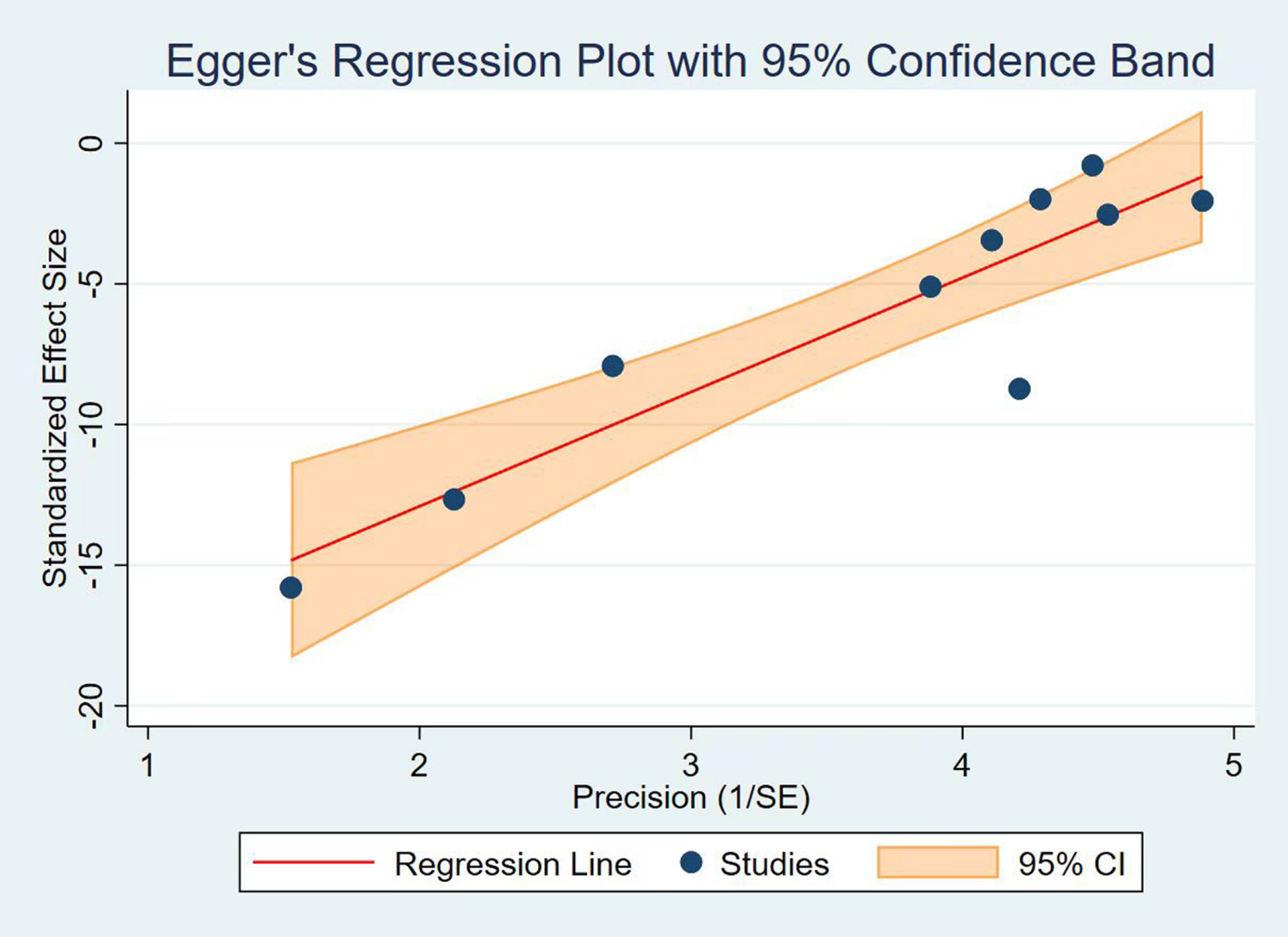


Fig. S17 Egger's regression plot of HbA1c
